# Parametric Estimations Based on Homomorphic Deconvolution for Time of Flight in Sound Source Localization System

**DOI:** 10.3390/s20030925

**Published:** 2020-02-10

**Authors:** Yeonseok Park, Anthony Choi, Keonwook Kim

**Affiliations:** 1 Division of Electronics & Electrical Engineering, Dongguk University-Seoul, Seoul 04620, Korea; dustjrdk@dongguk.edu; 2 Department of Electrical & Computer Engineering, Mercer University, 1501 Mercer University Drive, Macon, GA 31207, USA; choi_ta@mercer.edu

**Keywords:** sound source localization, time of flight, homomorphic deconvolution, cepstrum, Yule-walker, Prony, Steiglitz-McBride, vehicle

## Abstract

Vehicle-mounted sound source localization systems provide comprehensive information to improve driving conditions by monitoring the surroundings. The three-dimensional structure of vehicles hinders the omnidirectional sound localization system because of the long and uneven propagation. In the received signal, the flight times between microphones delivers the essential information to locate the sound source. This paper proposes a novel method to design a sound localization system based on the single analog microphone network. This article involves the flight time estimation for two microphones with non-parametric homomorphic deconvolution. The parametric methods are also suggested with Yule-walker, Prony, and Steiglitz-McBride algorithm to derive the coefficient values of the propagation model for flight time estimation. The non-parametric and Steiglitz-McBride method demonstrated significantly low bias and variance for 20 or higher ensemble average length. The Yule-walker and Prony algorithms showed gradually improved statistical performance for increased ensemble average length. Hence, the non-parametric and parametric homomorphic deconvolution well represent the flight time information. The derived non-parametric and parametric output with distinct length will serve as the featured information for a complete localization system based on machine learning or deep learning in future works.

## 1. Introduction

The sound source localization (SSL) system estimates the angle of arrival (AoA) for an acoustic source based on the received signal. SSL approaches are extensive, ranging from the physical rigid structures to the machine learning algorithms to design the spatial filter. The prevalent methods utilize the phase differences between the receivers for beamforming [1] which can be employed for such various applications as underwater warfare systems. Since the beamforming performance is proportional to the receiver quantity, numerous microphones are required for high precision AoA estimation. The beamforming constraints are challenged by the biomimetics methods. Humans can accurately localize sound sources in three-dimensional (3D) space by using the binaural correlation and structure profile. Various monaural and binaural sound localization systems have been proposed to imitate human-like hearing system [2,3,4,5,6,7]. Currently, researches are being conducted to understand the propagation on the dedicated structure with single or dual receivers in nature science and practical engineering.

Sound is the complementary to vision for navigating mobile objects. The acoustic information enhances the system safety since the sound can propagate over the obstacle by diffraction property to deliver the situation over the non-line-of-sight (NLOS) locations. Presently, the human and vehicle are cooperated to drive the transport sorely based on the vision information. Hence, indirect imminent endangerment cannot be realized until the human has the visual contact. For example, a car with emergency braking cannot be perceived by the indirect position observers. The squeal sound provides the situation. However, the system cannot recognize the direction to activate the pre-emptive safety devices which reduce or remove the impact of the secondary collisions. The sound information can be used for improving the safety of future autonomous transport system.

The driver barely obtains the acoustic information since the vehicle structure debilitates the propagation by airtight cabin. The acoustic perception on sound source and arrival direction are both required to understand the situation. The SSL system mounted on a vehicle could provide the comprehensive information to improve the driving conditions by monitoring the surroundings including the NLOS observation. The conventional SSL approaches recently employed for transport are as follows. The moving vehicle presences are identified by sound localization based on the arrival time difference between the microphones [8]. A sensing technique to localize an approaching vehicle is proposed by an acoustic cue from the spatial-temporal gradient method [9]. For the sequential movement events of vehicles, robust direction-of-arrival estimation is realized by the incoherent signal-subspace method based on a small microphone array [10]. 

**Figure 1:**
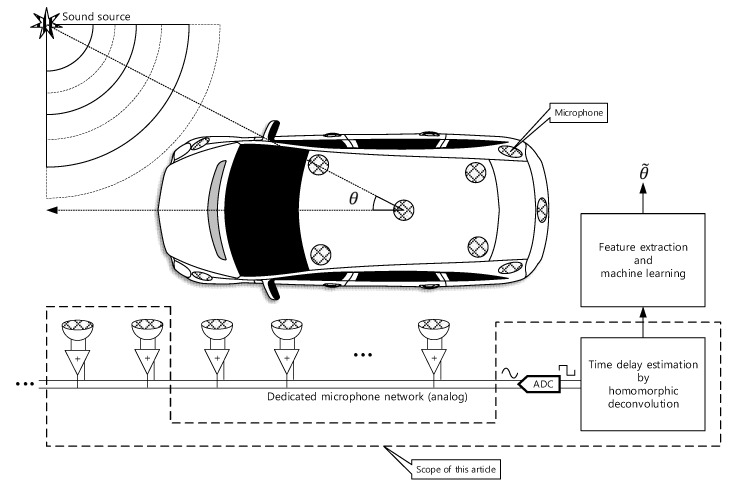
Functional diagram of overall SSL system for transport. This paper presents the parametric and non-parametric homomorphic deconvolution for two-microphones situation. The car shape is illustrated by Nichkov Alexey [13].

**Figure 2:**
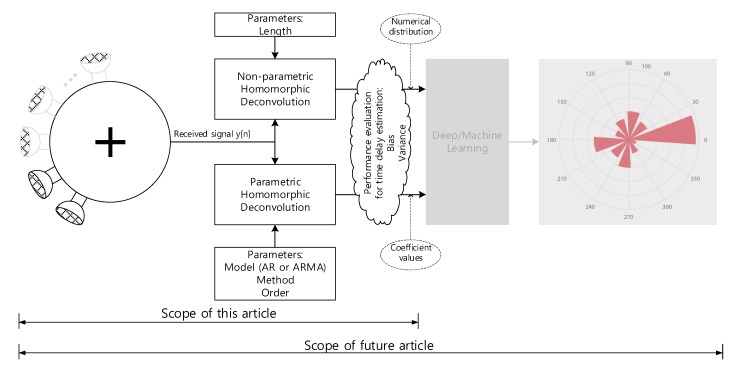
Overall methodology for vehicle sound source localization system. Note that the gray area will be investigated in the future article.

The 3D structure of vehicle with size causes the problems to realize the omnidirectional SSL system because of the long and uneven propagation. In addition, the aerodynamic profile of the vehicle prevents to install the monaural and binaural localizer which use the pinna-like configuration. This paper proposes the novel method to design the SSL system for transport based on the analog microphone network. The single channel signal utilizes the deconvolution process and machine learning (or deep learning) for SSL. The comprehensive functional diagram is illustrated in Figure 1. The mixed signal on the microphone network bus contains the various time delay information to represent the time of flight (ToF) between microphones. The implicit ToF is estimated by the homomorphic deconvolution (HD) algorithm, which is established through the homomorphic system [11,12]. In order to use the ToF on machine learning stage, the HD algorithm can be modified for parametric methods as extracted features. The coefficients of the parametric model as well as distribution of the non-parametric method are essential clues to derive the AoA information as shown in Figure 2. The suggested vehicle SSL system is extensive to describe the complete proposition in single article. Therefore, this paper only describes the ToF estimation for two microphones with non-parametric and parametric HD algorithms. Future articles will include the SSL performance with machine learning based on the findings in this paper.

Numerous investigations have been conducted for SSL system and are summarized below. Dong et al. [14] combine with the arrivals of multi-sensor and inversion of the real-time average for iterative method based on the time differences to locate source coordinates with clear arrivals. SSL monitors the development of the crack in real time for the complex structure by searching the fastest wave path over the structure with irregular spaces in order to improve location accuracy [15]. An analytical SSL method was developed for a simplified algorithm and conditions without an iterative algorithm, without pre-measured velocity, without initial value, and without square root operations [16]. The SSL system based on the embedded microphone array for outdoor environments is presented for unmanned aerial vehicle application [17]. The 3D sound source locations are estimated by generating direct and reflection acoustic rays based on ray tracing along with Monte Carlo localization algorithm [18]. Speech localization with microphone arrays is devised from the sparse Bayesian learning method by using a hierarchical two-level inference [19]. An extensive review and classification of sound source localization techniques and popular tracking methodologies is organized by Rascon and Meza [20].

The recent studies initiated to employ the machine learning methods for SSL systems are as follows. Observe that this paper focuses on the ToF estimation. The SSL system based on the ToF and machine learning which will be realized in the future article as shown in Figure 2. The reverberation robust feature extraction method for machine leaning is proposed by Li and Chen [21] for sound source localization based on sound intensity estimation over a microphone array. The sound localization system is designed from deep neural networks using the frequency domain feature based on the integrating directional information and directional activator [22]. For near-field sound localization, a weighted minimum variance distortionless response algorithm is presented with a machine learning for the steered response power computation [23]. The multiple source localization on underwater horizontal arrays is presented by using deep neural networks with two-stage architecture for the directions and ranges in shallow water [24].

This paper achieves the aims proposed by the authors’ previous SSL publications. The fundamental frequencies induced by the asymmetric horizontal pyramidal horns were arranged for the far-field monaural localization system by utilizing cepstral parameters [25]. The small-profile near-field monaural system was realized by the asymmetric vertical cylindrical pipes around a single microphone [26]. The reflective monaural localization system [27] placed the multiple plates for the direction-wise time delay to be estimated by homomorphic deconvolution. Other localization works on the subject by the authors are also related to and expanded during the research, such as azimuthal movement detection based on binaural architecture [28] and a target localization algorithm over a distributed acoustic sensor network [29]. Observe that the experiments are performed and evaluated within an identical anechoic chamber [30] to that used in the previous works.

## 2. Methodology

The ToF estimation for the two microphones can be described as the linear time-invariant (LTI) system with proper impulse response. The time distance between the receivers is contained over the two Kronecker delta functions (or delta functions) in the propagation impulse response. Without the noise condition, the sound source travels through the medium and last arrivals for microphones inscribe the time distance on the delta functions. The receiver structure presents the ToF based on the arrival angle; however, we assume that the ToF is given in this paper. As part of the sound localization, the system requires to obtain the ToF information with or without the sound source restoration. The received signal is the convolution sum (or integral) between the sound source and ToF impulse response. Therefore, the goal of this paper is the deconvolution problem between the two signals.

In order to realize the separation, the propagation function should be understood with parameters. The performance of the deconvolution depends on the signal and propagation condition since the deconvolution projects the problem domain into the maximally separable space. In this paper, the HD utilizes the homomorphic systems in cascade to remove the sound source and to derive the propagation function. The forward conversion of the homomorphic system is implemented by the real cepstrum to compress the geometric series from poles and zeros of the received signal model. In time domain, the propagation function shows the echo property as wide stride structure which provides relatively slow decreasing rate distribution after the real cepstrum. The distinct compression rates in cepstrum domain deliver the powerful tool to divide the signal and propagation. The simple window known as frequency-invariant linear filtering (FILF) performs the separation. The backward conversion of the homomorphic system by inverse cepstrum finally derives the propagation function for ToF. The real cepstrum extensively uses the discrete Fourier transform (DFT) or fast Fourier transform (FFT) for non-parametric estimation. The parametric ToF estimation is realized by the propagation function model with parametric estimation methods such as Yule-walker, Prony, and Steiglitz-McBride in last stage of HD. Note that the non-parametric technique produces the numerical distribution and parametric method estimates model coefficients. 

**Figure 3:**
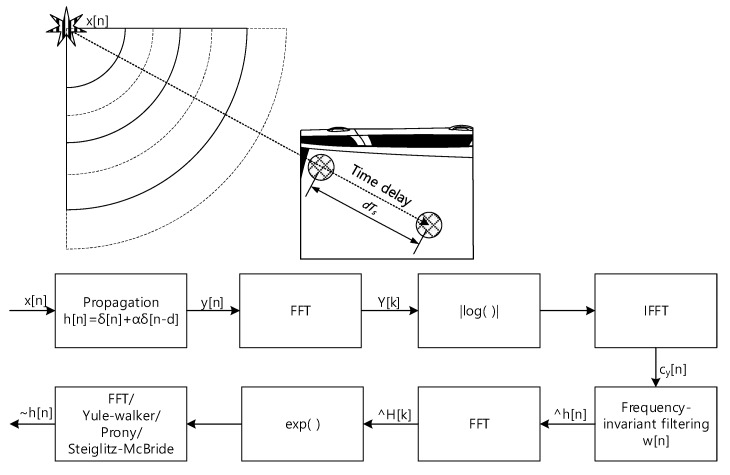
System architecture of time delay estimation based on the parametric and non-parametric homomorphic deconvolution.

Figure 3 demonstrates the overall signal propagation and estimation procedure. The wide or narrow band signal *x*[*n*] is delivered to the both microphone with *dT_s_* time difference. The *T_s_* is the sampling period and the corresponding propagation function *h*[*n*] is the δ[n]+αδ[n−d] with attenuation rate α. The received signal *y*[*n*] is the convolution sum (or integral) as x[n]∗h[n]. The first FFT and IFFT pair with absolute logarithm presents the real cepstrum to divide the signal and propagation distribution. The window function *w*[*n*] extracts the propagation function. The other FFT and IFFT pair with exponential function reestablishes and estimates the non-parametric propagation impulse response $\tilde{h}\left[n\right]$. The parametric estimation utilizes the regressive model to define the peaky distribution in propagation function as delta function. Originally, the estimation methods as Yule-walker, Prony, and Steiglitz-McBride is devised to present the spectral property of the signal. The parametric methods are applied to the estimation in the reverse direction. Therefore, the techniques should be extended by extension of signal and coefficient space into the complex number. The reverse usage will be explained in the following parametric section.

### 2.1. Homomorphic Deconvolution

The HD [31,32] in this paper consists of forward and backward real cepstrum to perform the deconvolution. The complex cepstrum [31,32] is shown in below. The $Y\left(e^{j\omega }\right)$ is the discrete-time Fourier transform (DTFT) of the received signal. The inverse DTFT of the complex logarithm on $Y\left(e^{j\omega }\right)$ is the complex cepstrum. The actual complex cepstrum is realized by the DFT which presents the principal values for phase. The additional procedure to unwarp the phase is necessary for proper cepstrum output. However, the complex cepstrum provides the capability to separate the sound source and propagation function approximately intact.

(1) $$\begin{array}{c} \hat{y}\left[n\right]=\frac{1}{2\pi }\int_{-\pi }^{\pi }\log\left(Y\left(e^{j\omega }\right)\right)e^{j\omega n}d\omega \end{array}$$

The real cepstrum [31,32] employs the absolute logarithm before the inverse DTFT as below. Because of the omitted phase information, the real cepstrum demonstrates the limited performance to obtain the sound source from the deconvolution. The deconvolution system estimates the ToF based on the propagation function. Hence, this paper exploits the real cepstrum for HD.

(2) $$c_{y}\left[n\right]=\frac{1}{2\pi }\int_{-\pi }^{\pi }\log\left|Y\left(e^{j\omega }\right)\right|e^{j\omega n}d\omega$$

The relationship between the complex and real cepstrum is below. Due to the absolute operation on the DTFT, the real cepstrum represents the even function property.

(3) $$c_{y}\left[n\right]=\frac{\hat{y}\left[n\right]+{\hat{y}}^{*}\left[-n\right]}{2}\;\;\;∵\;\left|Y\left(e^{j\omega }\right)\right|=\sqrt{Y\left(e^{j\omega }\right)Y^{*}\left(e^{j\omega }\right)}$$

The real cepstrum casts the sound source and propagation function on the distinct locations; therefore, the proper window can partition the propagation function from the other signals. Note that the sound source cannot be recovered from the real cepstrum in most of conditions. The *n* domain window *w*[*n*] is applied as below for FILF.

(4) $$\hat{h}\left[n\right]=c_{y}\left[n\right]w\left[n\right]$$

The inverse cepstrum is implemented by the inverse DTFT of the exponential $\hat{H}\left(e^{j\omega }\right)$ which is the $\tilde{h}\left[n\right]$ DTFT. The derived $\tilde{h}\left[n\right]$ corresponds to the propagation function; hence, the peak location except zero position determines the ToF. Observe that the cepstrum engages and disengages the domain by using the logarithm and exponential function with entrance and exit functions as Fourier and *Z*-transform. The analysis can be performed over the DTFT or *z* domain.

(5) $$\tilde{h}\left[n\right]=\frac{1}{2\pi }\int_{-\pi }^{\pi }e^{\hat{H}\left(e^{j\omega }\right)}e^{j\omega n}d\omega$$

The numerical realization by computer utilizes the DFT and IDFT for real cepstrum. The periodicity is developed as below due to the DFT and IDFT property. Also, the symmetricity is produced because of the absolute operation for logarithm. Note that *N* is the DFT length.

(6) $${\tilde{c}}_{y}\left[n\right]=\overset{\infty }{\underset{k=-\infty }{\sum }}c_{y}\left[n+kN\right];\;\;\;{\tilde{c}}_{y}\left[n\right]={\tilde{c}}_{y}^{*}\left[N-n\right]$$

The real cepstrum based on the DTFT denotes the infinite duration according to the inherited property. Therefore, the periodic ${\tilde{c}}_{y}\left[n\right]$ from the DFT is the time-aliased form of the $c_{y}\left[n\right]$. The increased DFT length *N* is recommended to avoid the data corruption due to the periodicity. The symmetricity plays the important role in the minimum phase system realization. The appropriate window *w*[*n*] should consider the ${\tilde{c}}_{y}\left[n\right]$ symmetricity for deconvolution based on the minimum- and maximum-phased decomposition. Appendix A delivers detailed analysis of the non-parametric HD with sound source and propagation model and example. 

### 2.2. Model Based Parametric Estimations

The parametric estimation is involved in the last stage of the inverse cepstrum. The estimation output is the coefficient values of desired signal model. The evaluation of the signal model with estimated coefficients corresponds to the propagation impulse response. The HD provides the deconvolution work and the parametric estimation presents the domain transfer. The conventional parametric spectral estimation algorithms require the frequency domain signal model as rational function. Therefore, following section explains the estimation algorithms for forward transformation. The parametric HD exercises the estimation algorithm for backward transformation that will be described in the last part of below section. The conventional signal models are below.

● Autoregressive (AR) model

(7) $$y\left[n\right]+a_{1}y\left[n-1\right]+\cdots +a_{N}y\left[n-N\right]=x\left[n\right]$$

(8) $$H\left(z\right)=\frac{1}{1+a_{1}z^{-1}+\cdots +a_{N}z^{-N}}$$

● Autoregressive moving average (ARMA) model

(9) $$y\left[n\right]+a_{1}y\left[n-1\right]+\cdots +a_{N}y\left[n-N\right]=b_{0}x\left[n\right]+b_{1}x\left[n-1\right]+\cdots +b_{M}x\left[n-M\right]$$

(10) $$H\left(z\right)=\frac{b_{0}+b_{1}z^{-1}+\cdots +b_{M}z^{-M}}{1+a_{1}z^{-1}+\cdots +a_{N}z^{-N}}$$

The signal model is chosen by comprehending the desired response profile. The AR model uses the poles and the ARMA model utilizes the zeros and poles at *z* domain. The zeros close to the unit circle presents the valley shape distribution and the poles nearby the unit circle provides the peaky style response in the domain. Observe that group of poles or zeros could demonstrate the flat with slight fluctuation response. After the signal model is selected, the order and coefficients are estimated by the various algorithms. The estimation process requires the statistical signal processing which regards signals as stochastic processes. The expectation, (auto)covariance, etc. operations based on the stationary white noise are exercised with various assumptions. 

The following is the fundamental requirements and operations for statistical signal processing. The signal *y*[*n*] is necessary to be a random variable sequence with zero mean as below.

(11) E{y[n]}=0   for all n∈ℤ

where the *E*{ } represents the expectation operation over the ensemble realizations. The auto-covariance sequence (ACS) is presented as below.

(12) $$r\left[k\right]=E\left\{y\left[n\right]y^{*}\left[n-k\right]\right\}$$

With above conditions, the signal *y*[*n*] is a second-order stationary sequence. Due to the limited length of the signal, the biased estimation of ACS is below.

(13) $$\tilde{r}\left[k\right]=\frac{1}{N}\overset{N}{\underset{n=k+1}{\sum }}y\left[n\right]y^{*}\left[n-k\right]\;\;\;\;\mathrm{for}\;0\leq k\leq N-1\;\mathrm{and}\;1\leq n\leq N$$

The biased ACS estimate is usually used for the processing because the biased $\tilde{r}\left[k\right]$ sequence is guaranteed to be positive semidefinite for positive spectral estimation [33].

#### 2.2.1. Yule-Walker (AR Model Based)

The Yule-Walker algorithm [34] figures out signal distribution based on the AR model. Below is the conventional AR model in time domain with input *e*[*n*] which is the white noise with $\sigma^{2}$ variance.

(14) $$y\left[n\right]+a_{1}y\left[n-1\right]+\cdots +a_{N}y\left[n-N\right]=e\left[n\right]$$

Apply the $y^{*}\left[n-k\right]$ product and expectation in both side of the equation as below.

(15) $$E\left\{y^{*}\left[n-k\right]\left(y\left[n\right]+a_{1}y\left[n-1\right]+\cdots +a_{N}y\left[n-N\right]\right)\right\}=E\left\{y^{*}\left[n-k\right]e\left[n\right]\right\}$$

By definition of ACS, the left-hand side of the equation is modified as below.

(16) $$r\left[k\right]+\overset{N}{\underset{j=1}{\sum }}a_{j}r\left[k-j\right]=E\left\{y^{*}\left[n-k\right]e\left[n\right]\right\}$$

The right-hand side of the equation is determined by the *k* value condition. For the positive *k* value, the $y^{*}\left[n-k\right]$ and e[n] is uncorrelated; hence, the expectation is zero as below.

(17) $$r\left[k\right]+\overset{N}{\underset{j=1}{\sum }}a_{j}r\left[k-j\right]=0\;\;\;\mathrm{for}\;k>0$$

For zero *k* value, the right-hand side of the equation is equivalent to the $E\left\{e^{*}\left[n\right]e\left[n\right]\right\}$ as $\sigma^{2}$ due to the zero mean white noise *e*[*n*].

(18) $$r\left[0\right]+\overset{N}{\underset{j=1}{\sum }}a_{j}r\left[-j\right]=\sigma^{2}\;\;\;\;\;\mathrm{for}\;k=0\;\;r\left[k\right]=r^{*}\left[-k\right]$$

Equations (17) and (18) are organized in matrix form as below. The lines in the following matrix are segmentation boundaries for submatrix construction. Equation (19) is known as the Yule-Walker equation or Normal equation to develop the fundamental of many AR estimation methods.

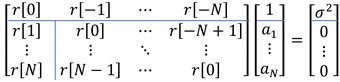
(19)

The submatrix is demonstrated as below. The solution of the following equation provides the coefficient estimation. However, computational limitation cannot realize the equation directly.

(20) $$\left[\begin{array}{ccc} r\left[0\right] & \cdots & r\left[-N+1\right]\\ \vdots & \ddots & \vdots \\ r\left[N-1\right] & \cdots & r\left[0\right] \end{array}\right]\left[\begin{array}{c} a_{1}\\ \vdots \\ a_{N} \end{array}\right]=-\left[\begin{array}{c} r\left[1\right]\\ \vdots \\ r\left[N\right] \end{array}\right]$$

The biased ACS estimation with limited length data presents the below equation for the feasible approach. Note that the square matrix for inversion operation is Toeplitz matrix.

(21) $$\left[\begin{array}{c} {\tilde{a}}_{1}\\ \vdots \\ {\tilde{a}}_{N} \end{array}\right]=-{\left[\begin{array}{ccc} \tilde{r}\left[0\right] & \cdots & \tilde{r}\left[-N+1\right]\\ \vdots & \ddots & \vdots \\ \tilde{r}\left[N-1\right] & \cdots & \tilde{r}\left[0\right] \end{array}\right]}^{-1}\left[\begin{array}{c} \tilde{r}\left[1\right]\\ \vdots \\ \tilde{r}\left[N\right] \end{array}\right]$$

Based on the calculated coefficients, the spectral distribution can be represented by the subsequent rational function from DTFT.

(22) $${\left|\tilde{H}\left(e^{j\omega }\right)\right|}^{2}=\frac{\sigma^{2}}{{\left|1+{\tilde{a}}_{1}e^{-j\omega }+\cdots +{\tilde{a}}_{N}e^{-j\omega N}\right|}^{2}}$$

This paper assumes that the order of the estimation methods is given based on the signal condition.

#### 2.2.2. Prony (ARMA Model Based)

The Prony algorithm [35] estimates the coefficients of ARMA model for various types of signal. The complexity is developed from the nonlinear property of the ARMA difference equation. The impulse response of the ARMA model linearizes the system to provide the matrix description. The solution of the matrix derives the parameter estimation for ARMA. Below is the ARMA model in difference equation form.

(23) $$y\left[n\right]+a_{1}y\left[n-1\right]+\cdots +a_{N}y\left[n-N\right]=b_{0}x\left[n\right]+\cdots +b_{M}x\left[n-M\right]$$

The rational function in *z* domain presents the impulse response as below.

(24) $$\frac{Y\left(z\right)}{X\left(z\right)}=\frac{b_{0}+b_{1}z^{-1}+\cdots +b_{M}z^{-M}}{1+a^{1}z^{-1}+\cdots +a_{N}z^{-N}}=\frac{B\left(z\right)}{A\left(z\right)}=H\left(z\right)=h_{0}+h_{1}z^{-1}+h_{2}z^{-2}+\cdots$$

Note that the impulse response *h*[*n*] is most likely to be infinite length sequence because of the rational function property. The product between the impulse response and denominator demonstrates the numerator of the rational function in *z* domain as below.

(25) B(z)=H(z)A(z)

Observe that the length of *a* and *b* coefficients are finite. However, the *h* length is infinite in general as below.

(26) $$b_{0}+b_{1}z^{-1}+\cdots +b_{M}z^{-M}=\left(h_{0}+h_{1}z^{-1}+h_{2}z^{-2}+\cdots \right)\left(1+a^{1}z^{-1}+\cdots +a_{N}z^{-N}\right)$$

The above polynomial in *z* domain can be organized in matrix form as below. The lines in the following matrix are segmentation boundaries for submatrix construction.

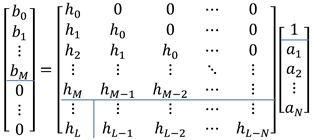
(27)

The submatrix is demonstrated in bold alphabet as below. The upper and lower case represent the matrix and vector, respectively.

(28) $$\left[\frac{b}{0}\right]=\left[\frac{H_{1}}{\begin{array}{cc} h_{1} & H_{2} \end{array}}\right]\left[\frac{1}{a^{†}}\right]$$

The solution of the following equations provides the coefficient estimations as $\tilde{a}$ and $\tilde{b}$. Note that $a^{†}$ is the submatrix of ***a*** column vector from the second row to end.

(29) $$0=h_{1}+H_{2}a^{†}$$

$$b=H_{1}a$$

Based on the calculated coefficients, the spectral distribution can be represented by the subsequent rational function from DTFT.

(30) $${\left|\tilde{H}\left(e^{j\omega }\right)\right|}^{2}=\frac{{\left|{\tilde{b}}_{0}+{\tilde{b}}_{1}e^{-j\omega }+\cdots +{\tilde{b}}_{M}e^{-j\omega M}\right|}^{2}}{{\left|1+{\tilde{a}}_{1}e^{-j\omega }+\cdots +{\tilde{a}}_{N}e^{-j\omega N}\right|}^{2}}$$

Therefore, the Prony receives the *h*[*n*] as the input to estimate the *a* and *b* coefficients for describing the unknown system model in best. Note that the longer ***H*** matrix could lead the solution to the least square sense due to the non-square matrix size.

#### 2.2.3. Steiglitz-McBride (ARMA Model Based)

**Figure 4:**
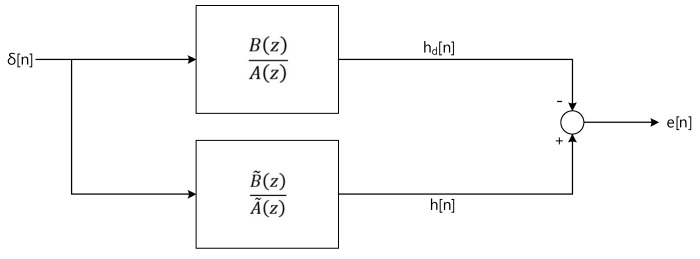
System model for Stiglitz-McBride algorithm.

The Steiglitz-McBride method [36] is the iterative technique to identify an ARMA system by minimizing the mean square error between the system and model output. Figure 4 shows the simplified system model for the Steiglitz-McBride algorithm.

The Steiglitz-McBride method recognizes the target system *B*(*z*)/*A*(*z*) by minimizing the energy of *e*[*n*] to equalize the model system $\tilde{B}\left(z\right)/\tilde{A}\left(z\right)$ to the target. The impulse response of the target and model are denoted by *h_d_*[*n*] and *h*[*n*], respectively. The system error *e*[*n*] is represented as below.

(31) $$e\left[n\right]=h_{d}\left[n\right]-h\left[n\right]\overset{Z}{↔}E\left(z\right)=\frac{B\left(z\right)}{A\left(z\right)}-\frac{\tilde{B}\left(z\right)}{\tilde{A}\left(z\right)}$$

The solution is given as below equation. The arguments of the minima are the points of the function at which the error *e*[*n*] energy is minimized. Therefore, the difference between the target and model system is decreased to the least.

(32) $$\tilde{\gamma }=\underset{\gamma }{\mathrm{argmin}}\underset{n}{\sum }{\left|e\left[n\right]\right|}^{2}\;\;\;\mathrm{where}\;\gamma =\left\{a_{1},\ldots ,\;a_{N},\;b_{0},\;\ldots ,\;b_{M}\right\}\;\mathrm{from}\;\frac{\tilde{B}\left(z\right)}{\tilde{A}\left(z\right)}$$

**Figure 5:**
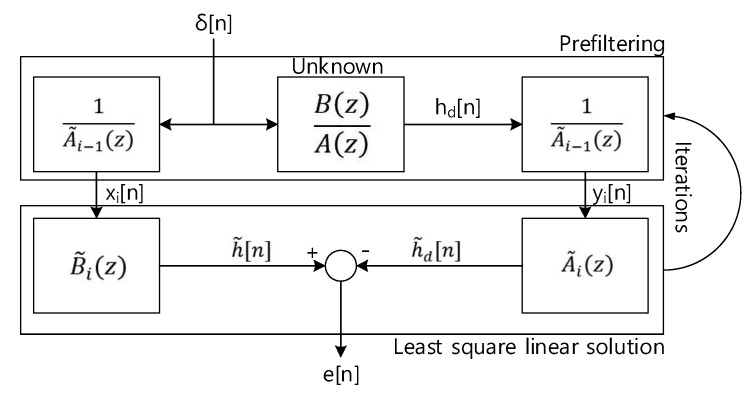
Modified system identification model for Stiglitz-McBride algorithm.

The rational function of ARMA provides the recursive equation; hence, the solution of previous equation implies the highly nonlinear and intractable property. The modified system model as Figure 5 presents the linearized solution by iterative approach.

Subsequent equations show the proof of equivalence for system identification problem between the target and model. As the error norm approaches to zero, below equation demonstrates that the target and model system are became identical according to the Parseval’s theorem.

(33) $$\underset{n}{\sum }{\left|e\left[n\right]\right|}^{2}=\frac{1}{2\pi j}\oint_{c}^{}{\left|H_{d}\left(z\right)\frac{{\tilde{A}}_{i}\left(z\right)}{{\tilde{A}}_{i-1}\left(z\right)}-\frac{{\tilde{B}}_{i}\left(z\right)}{{\tilde{A}}_{i-1}\left(z\right)}\right|}^{2}z^{-1}dz=\frac{1}{2\pi j}\oint_{c}^{}{\left|H_{d}\left(z\right){\tilde{A}}_{i}\left(z\right)-{\tilde{B}}_{i}\left(z\right)\right|}^{2}{\left|\frac{1}{{\tilde{A}}_{i-1}\left(z\right)}\right|}^{2}z^{-1}dz$$

Hence, the optimal numerator and denominator coefficients can be derived by solving the below equation. The ${\tilde{A}}_{i-1}\left(z\right)$ and ${\tilde{A}}_{i}\left(z\right)$ are mutually related. Therefore, the iterative method should be employed.

(34) $$\begin{array}{c} \tilde{\gamma }=\underset{\gamma }{\mathrm{argmin}}\underset{n}{\sum }{\left|e\left[n\right]\right|}^{2}=\underset{\gamma }{\mathrm{argmin}}\oint_{c}^{}{\left|H_{d}\left(z\right){\tilde{A}}_{i}\left(z\right)-{\tilde{B}}_{i}\left(z\right)\right|}^{2}{\left|\frac{1}{{\tilde{A}}_{i-1}\left(z\right)}\right|}^{2}z^{-1}dz\\ =\underset{\gamma }{\mathrm{argmin}}\oint_{c}^{}{\left|H_{d}\left(z\right){\tilde{A}}_{i}\left(z\right)-{\tilde{B}}_{i}\left(z\right)\right|}^{2}z^{-1}dz \end{array}$$

As shown in Equation (33) and Figure 5, Equation (34) is converted to the linear problem as ${\left|H_{d}\left(z\right){\tilde{A}}_{i}\left(z\right)-{\tilde{B}}_{i}\left(z\right)\right|}^{2}$ by the prefiltering with $1/{\tilde{A}}_{i-1}\left(z\right)$. The prefiltering with desired response and delta function are given as below.

(35) $$x_{i}\left[n\right]=-a_{\left(i-1\right)1}x_{i}\left[n-1\right]-a_{\left(i-1\right)2}x_{i}\left[n-2\right]-\cdots -a_{\left(i-1\right)N}x_{i}\left[n-M\right]+\delta \left[n\right]$$

(36) $$y_{i}\left[n\right]=-a_{\left(i-1\right)1}y_{i}\left[n-1\right]-a_{\left(i-1\right)2}y_{i}\left[n-2\right]-\cdots -a_{\left(i-1\right)N}y_{i}\left[n-M\right]+h_{d}\left[n\right]$$

The prefiltering part of Figure 5 is demonstrated by Equations (35) and (36) for left-hand and right-hand side. The matrix representations below stand for the convolution sum. The least square linear solution part of Figure 5 is denoted by the below two matrix operations for left-hand and right-hand side.

(37) $$\left[\begin{array}{ccccc} x_{i}\left[0\right] & 0 & 0 & \cdots & 0\\ x_{i}\left[1\right] & x_{i}\left[0\right] & 0 & \cdots & 0\\ x_{i}\left[2\right] & x_{i}\left[1\right] & x_{i}\left[0\right] & \cdots & 0\\ \vdots & \vdots & \vdots & \ddots & \vdots \\ x_{i}\left[M\right] & x_{i}\left[M-1\right] & x_{i}\left[M-2\right] & \cdots & x_{i}\left[0\right]\\ x_{i}\left[M+1\right] & x_{i}\left[M\right] & x_{i}\left[M-1\right] & \cdots & x_{i}\left[1\right]\\ \vdots & \vdots & \vdots & \ddots & \vdots \\ x_{i}\left[L\right] & x_{i}\left[L-1\right] & x_{i}\left[L-2\right] & \cdots & x_{i}\left[L-M\right] \end{array}\right]\left[\begin{array}{c} b_{i0}\\ b_{i1}\\ b_{i2}\\ \vdots \\ b_{iM} \end{array}\right]=\left[\begin{array}{c} {\tilde{h}}_{i}\left[0\right]\\ {\tilde{h}}_{i}\left[1\right]\\ {\tilde{h}}_{i}\left[2\right]\\ \vdots \\ {\tilde{h}}_{i}\left[L\right] \end{array}\right]$$

(38) $$\left[\begin{array}{ccccc} y_{i}\left[0\right] & 0 & 0 & \cdots & 0\\ y_{i}\left[1\right] & y_{i}\left[0\right] & 0 & \cdots & 0\\ y_{i}\left[2\right] & y_{i}\left[1\right] & y_{i}\left[0\right] & \cdots & 0\\ \vdots & \vdots & \vdots & \ddots & \vdots \\ y_{i}\left[N\right] & y_{i}\left[N-1\right] & y_{i}\left[N-2\right] & \cdots & y_{i}\left[0\right]\\ y_{i}\left[N+1\right] & y_{i}\left[N\right] & y_{i}\left[N-1\right] & \cdots & y_{i}\left[1\right]\\ \vdots & \vdots & \vdots & \ddots & \vdots \\ y_{i}\left[L\right] & y_{i}\left[L-1\right] & y_{i}\left[L-2\right] & \cdots & y_{i}\left[L-N\right] \end{array}\right]\left[\begin{array}{c} a_{i0}\\ a_{i1}\\ a_{i2}\\ \vdots \\ a_{iN} \end{array}\right]=\left[\begin{array}{c} {\tilde{h}}_{d}{}_{i}\left[0\right]\\ {\tilde{h}}_{d}{}_{i}\left[1\right]\\ {\tilde{h}}_{d}{}_{i}\left[2\right]\\ \vdots \\ {\tilde{h}}_{d}{}_{i}\left[L\right] \end{array}\right]$$

For zero error norm condition, the above equations should be equal as below.

(39) $${\tilde{h}}_{i}\left[n\right]={\tilde{h}}_{d}{}_{i}\left[n\right]\;\;\mathrm{for}\;e_{i}\left[n\right]=0\;\mathrm{and}\;0\leq n\leq L$$

With zero error norm condition, the above matrix can be combined as below.

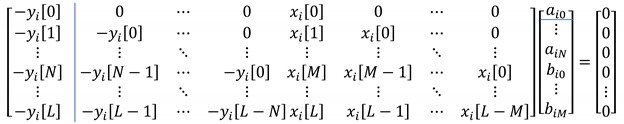
(40)

The first column of the matrix and first row of the column vector are partitioned and reorganized as below.

(41) $$\left[\begin{array}{ccc} 0 & \cdots & 0\\ -y_{i}\left[0\right] & \cdots & 0\\ \vdots & \ddots & \vdots \\ -y_{i}\left[N-1\right] & \cdots & -y_{i}\left[0\right]\\ \vdots & \ddots & \vdots \\ -y_{i}\left[L-1\right] & \cdots & -y_{i}\left[L-N\right] \end{array}\begin{array}{cccc} x_{i}\left[0\right] & 0 & \cdots & 0\\ x_{i}\left[1\right] & x_{i}\left[0\right] & \cdots & 0\\ \vdots & \vdots & \ddots & \vdots \\ x_{i}\left[M\right] & x_{i}\left[M-1\right] & \cdots & x_{i}\left[0\right]\\ \vdots & \vdots & \ddots & \vdots \\ x_{i}\left[L\right] & x_{i}\left[L-1\right] & \cdots & x_{i}\left[L-M\right] \end{array}\right]\left[\begin{array}{c} a_{i1}\\ \vdots \\ a_{iN}\\ b_{i0}\\ \vdots \\ b_{iM} \end{array}\right]=\left[\begin{array}{c} y_{i}\left[0\right]\\ y_{i}\left[1\right]\\ \vdots \\ y_{i}\left[N\right]\\ \vdots \\ y_{i}\left[L\right] \end{array}\right]$$

The short representation of above matrix is below. Note that the *i* in the subscript indicates the *i*-th iterations.

(42) $$H_{i}c_{i}=y_{i}$$

The optimal parameters can be obtained by solving the linear solver such as QR solver to find minimum norm residual solution.

(43) $$c_{i}=\mathrm{Linear}\;\mathrm{Solver}\left(H_{i},y_{i}\right)$$

Repeat the procedures for several times. Final ***c_i_*** is the optimal $\tilde{c}$. In general, the iterative method converges to the optimal solution rapidly. The Prony method provides the good initial estimation of a_(0)*i*_ coefficients for Equation (35) and Equation (36). Based on the calculated coefficients $\tilde{c}$, the spectral distribution can be represented by the subsequent rational function from DTFT.

(44) $${\left|\tilde{H}\left(e^{j\omega }\right)\right|}^{2}=\frac{{\left|{\tilde{b}}_{0}+{\tilde{b}}_{1}e^{-j\omega }+\ldots +{\tilde{b}}_{M}e^{-j\omega M}\right|}^{2}}{{\left|1+{\tilde{a}}_{1}e^{-j\omega }+\ldots +{\tilde{a}}_{N}e^{-j\omega N}\right|}^{2}}$$

Therefore, the Steiglitz-McBride algorithm receives the *h_d_*[*n*] as the input to estimate the prefiltering coefficients by using the Prony algorithm first. After the prefiltering, the linear solver generates the numerator and denominator coefficients. Repeat the prefiltering and linear solving until the error norm reaches to the minimum requirements. 

## 3. Simulations

The non-parametric and parametric HD algorithms are theoretically explained at previous sections. In order to summarize the algorithm sequence, below equations are organized in the chronological order. Note that the numerical realizations by computer require the DFT (or FFT) for domain transfer. The *Y*[*k*] is the FFT of the received signal with the power of two in order length *L*. The real cepstrum is realized as below.

(45) $$c_{y}\left[n\right]=\frac{1}{N}\overset{N-1}{\underset{k=0}{\sum }}\log\left|Y\left[k\right]\right|e^{j\frac{2\pi }{N}kn}$$

Apply the FILF for minimum phase realization and low time delay removal. The FILF functions are described at Appendix A in detail.

(46) $$\hat{h}\left[n\right]=c_{y}\left[n\right]w\left[n\right]$$

The $e^{\hat{H}\left[k\right]}$ denotes the exponential of $\hat{h}\left[n\right]$ FFT with length *L*. To maintain the consistency, forward FFT is applied to obtain the inverse transformation as below. The input conjugate to the FFT provides the conjugated inverse FFT outcome as below.

(47) $$\tilde{h}\left[n\right]=\frac{1}{L}\overset{L-1}{\underset{k=0}{\sum }}e^{\hat{H}\left[k\right]}e^{j\frac{2\pi }{L}kn}=\frac{1}{L}{\left\{\overset{L-1}{\underset{k=0}{\sum }}{\left(e^{\hat{H}\left[k\right]}\right)}^{*}e^{-j\frac{2\pi }{L}kn}\right\}}^{*}=\frac{1}{L}{\left\{{\mathrm{FFT}}_{L}{\left(e^{\hat{H}\left[k\right]}\right)}^{*}\right\}}^{*}$$

Since the $\tilde{h}\left[n\right]$ magnitude corresponds to the time delay distribution, the absolute of scaled version as below represents the second microphone position by ToF. The maximum magnitude position of $\left|{\tilde{h}}_{HD}\left[n\right]\right|$ demonstrates the time delay between the microphones.

(48) $$\left|{\tilde{h}}_{HD}\left[n\right]\right|=\frac{1}{L}\left|{\mathrm{FFT}}_{L}{\left(e^{\hat{H}\left[k\right]}\right)}^{*}\right|$$

The parametric HD follows the exactly identical procedures until $e^{\hat{H}\left[k\right]}$ computation. The Yule-walker, Prony, and Steiglitz-McBride algorithms are originally devised for forward domain transfer; therefore, the input conjugate to the parametric algorithms generate the conjugated parameters for estimation. Below equation is illustrated for Yule-walker method.

(49) $$\left\{{\tilde{a}}_{1},{\tilde{a}}_{2}\ldots ,\;{\tilde{a}}_{N},\;{\tilde{\sigma }}^{2}\right\}=\mathrm{YW}\left({\left(e^{\hat{H}\left[k\right]}\right)}^{*}\right)$$

Because the ${\tilde{h}}_{\mathrm{YW}}\left[n\right]$ magnitude shows the time delay distribution, the absolute of rational function as below presents the ToF. The maximum magnitude location in *n* of $\left|{\tilde{h}}_{\mathrm{YW}}\left[n\right]\right|$ indicates the time delay between the microphones.

(50) $${\left|{\tilde{h}}_{\mathrm{YW}}\left[n\right]\right|}^{2}=\frac{{\tilde{\sigma }}^{2}}{{\left|1+{\tilde{a}}_{1}e^{-j\frac{2\pi }{L}n}+{\tilde{a}}_{2}e^{-j2\frac{2\pi }{L}n}+\ldots +{\tilde{a}}_{N}e^{-jN\frac{2\pi }{L}n}\right|}^{2}}\;\;\;\;\left\{{\tilde{a}}_{1},{\tilde{a}}_{2}\ldots ,\;{\tilde{a}}_{N}\right\}\in \mathbb{C};\;0\leq n\leq L-1$$

Below equation is illustrated for Prony method.

(51) $$\left\{{\tilde{a}}_{1},\ldots ,\;{\tilde{a}}_{N},\;{\tilde{b}}_{0},\;\ldots ,\;{\tilde{b}}_{M}\right\}=\mathrm{Prony}\left({\left(e^{\hat{H}\left[k\right]}\right)}^{*}\right)$$

The maximum magnitude location in *n* of $\left|{\tilde{h}}_{\mathrm{Prony}}\left[n\right]\right|$ indicates the ToF between the microphones.

(52) $${\left|{\tilde{h}}_{\mathrm{Prony}}\left[n\right]\right|}^{2}=\frac{{\left|{\tilde{b}}_{0}+{\tilde{b}}_{1}e^{-j\frac{2\pi }{L}n}+\ldots +{\tilde{b}}_{M}e^{-jM\frac{2\pi }{L}n}\right|}^{2}}{{\left|1+{\tilde{a}}_{1}e^{-j\frac{2\pi }{L}n}+\ldots +{\tilde{a}}_{N}e^{-jN\frac{2\pi }{L}n}\right|}^{2}}\;\;\;\left\{{\tilde{a}}_{1},\ldots ,\;{\tilde{a}}_{N},\;{\tilde{b}}_{0},\;\ldots ,\;{\tilde{b}}_{M}\right\}\in \mathbb{C};\;\;0\leq n\leq L-1$$

Below equation displays the Steiglitz-McBride method.

(53) $$\left\{{\tilde{a}}_{1},\ldots ,\;{\tilde{a}}_{N},\;{\tilde{b}}_{0},\;\ldots ,\;{\tilde{b}}_{M}\right\}=\mathrm{StMc}\left({\left(e^{\hat{H}\left[k\right]}\right)}^{*}\right)$$

The maximum magnitude location in *n* of $\left|{\tilde{h}}_{\mathrm{StMc}}\left[n\right]\right|$ delivers the ToF between the microphones.

(54) $${\left|{\tilde{h}}_{\mathrm{StMc}}\left[n\right]\right|}^{2}=\frac{{\left|{\tilde{b}}_{0}+{\tilde{b}}_{1}e^{-j\frac{2\pi }{L}n}+\ldots +{\tilde{b}}_{M}e^{-jM\frac{2\pi }{L}n}\right|}^{2}}{{\left|1+{\tilde{a}}_{1}e^{-j\frac{2\pi }{L}n}+\ldots +{\tilde{a}}_{N}e^{-jN\frac{2\pi }{L}n}\right|}^{2}}\;\;\;\left\{{\tilde{a}}_{1},\ldots ,\;{\tilde{a}}_{N},\;{\tilde{b}}_{0},\;\ldots ,\;{\tilde{b}}_{M}\right\}\in \mathbb{C};\;\;0\leq n\leq L-1$$

The data used for the simulation is generated from the standard Gaussian distribution with zero mean and unit variance. Two independent and identically distributed random variables as $e_{s}\left[n\right]$ and $e_{m}\left[n\right]$ correspond to the signal and noise source, respectively as below.

(55) $$e_{s}\left[n\right]\sim N\left(0,1\right)\;\;\mathrm{and}\;e_{m}\left[n\right]\sim N\left(0,1\right)$$

The sound source *x*[*n*] is provided by the filtering the *e_s_*[*n*] with infinite impulse response (IIR) filter based on the Butterworth algorithm as below. The IIR_LPF_ function generates the corresponding coefficients and filter function performs the filter operation in time domain with coefficients and input data *e_s_*[*n*]. The $\omega_{c}$ is the cutoff frequency which represents the half magnitude in filter response. Further, 10 is the filter order.

(56) $$x\left[n\right]=\mathrm{filter}\left({\mathrm{IIR}}_{\mathrm{LPF}}\left(10,\omega_{c}\right),e_{s}\left[n\right]\;\right)$$

The received signal *y*[*n*] is produced with scaled and delayed signal and independent noise as below. The *d* is the delay in samples and α is the signal attenuation factor for second receiver. The β is the noise control factor.

(57) $$y\left[n\right]=x\left[n\right]+\alpha x\left[n-d\right]+\alpha \beta e_{m}\left[n\right]$$

Due to the general physics basis, the α magnitude is less than one; however, the β magnitude can be greater than one to control the signal-to-noise ratio (SNR) which is given as below. Note that the variance of sound source *x*[*n*] is equivalent to the cutoff frequency $\omega_{c}$ with given source $e_{s}\left[n\right]$.

(58) $$\mathrm{SNR}\;\left(\mathrm{dB}\right)=10\log_{10}\left(\frac{\omega_{c}+\alpha^{2}\omega_{c}}{{\left(\alpha \beta \right)}^{2}}\right)$$

The desired SNR can be achieved by adopting the following equation. The given SNR computes the required β value for noise magnitude. Also, the signal attenuation factor α is fixed for 0.8 in this simulation.

(59) $$\beta =\sqrt{\frac{\omega_{c}+\alpha^{2}\omega_{c}}{\alpha^{2}10^{\frac{SNR}{10}}}}$$

Once the received signal *y*[*n*] is created from the given and derived parameters, the non-parametric and parametric HD algorithms can improve the SNR by using the ensemble average. The average over the framed data *y_k_*[*n*] enhances the noise variance in inversely proportional manner as shown below. Note that the average is performed at the identical time positions over the frames.

(60) $$y\left[n\right]=\frac{1}{R}\overset{R-1}{\underset{k=0}{\sum }}y_{k}\left[n\right]\;\;\to \;\sigma_{y}^{2}\approx \frac{\sigma_{y_{k}}^{2}}{R}$$

**Figure 6:**
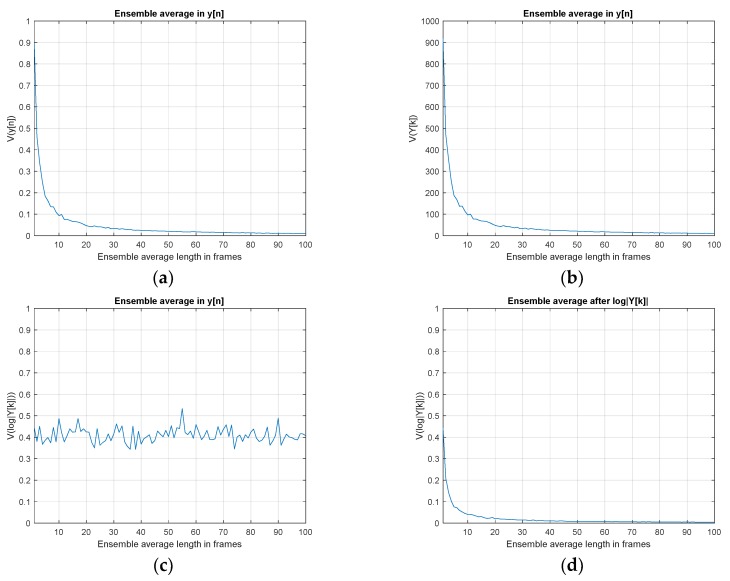
Signal variance at each computational stage of HD algorithm with pure noise situation as y[n]~N(0,1). The frame size is 1024 samples: (**a**) variance of *y*[*n*] with ensemble averaged *y*[*n*]; (**b**) variance of *Y*[*k*] with ensemble averaged *y*[*n*]; (**c**) variance of log|*Y*[*k*]| with ensemble averaged *y*[*n*]; (**d**) variance of log|*Y*[*k*]| over ensemble averaged log|*Y*[*k*]|.

The ensemble average maintains the signal power and reduces the noise variance for increased SNR over conventional linear operations. However, the non-linear operations such as logarithm could show the strong narrow proportionality for the average. Figure 6 demonstrates the variances of HD algorithm up to log|Y[k]| stage for various ensemble average length *R*. The received signal *y*[*n*] is pure noise based on the standard Gaussian distribution with zero mean and unit variance. The ensemble averaged *y*[*n*] delivers the inversely proportional variance for *y*[*n*] and *Y*[*k*] against the ensemble length. However, the variance of log|Y[k]| sustains the constant value with fluctuation due to the non-linearity of logarithm operation. Therefore, instead of taking the average on the *y*[*n*], performing the average after log|Y[k]| presents the inverse proportionality in variance which is illustrated in the last figure of below. 

Based on the previously stated signal model and ensemble average, the received signal is generated for simulation. The FILF window *w*[*n*] is determined to remove the 40 sample below and maximum phase realization. The detail *w*[*n*] function and performance can be observed in Appendix A. In this paper, the computed non-parametric and parametric HD output $\tilde{h}\left[n\right]$ is normalized to explore the maximum location *n* which corresponds to the ToF. Note that the non-parametric HD demonstrates the distribution by direct values. The parametric HD should evaluate the rational functions given in Equations (50), (52), and (54) with derived coefficient values from Yule-Walker, Prony, and Steiglitz-McBride. 

**Figure 7:**
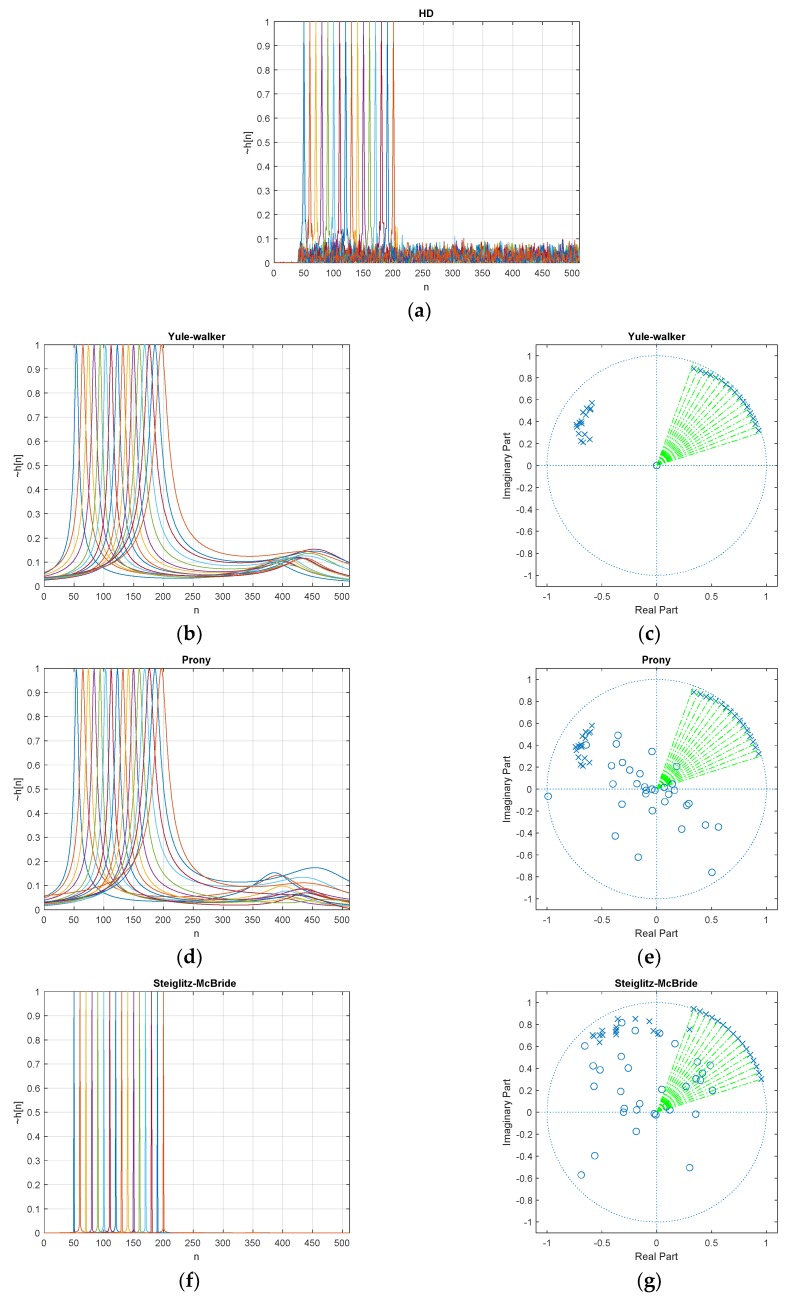
Estimated $\tilde{h}\left[n\right]$ distribution (normalized) and pole-zero plot with 20 dB SNR & 20 ensemble average for ToF (50~200 in 10 sample interval) from: (**a**) non-parametric HD; (**b**) Yule-walker HD; (**c**) corresponding pole-zero plot; (**d**) Prony HD; (**e**) corresponding pole-zero plot; (**f**) Steiglitz-McBride HD; (**g**) corresponding pole-zero plot. The pole/zero locations are indicated by x/o mark, respectively. The green dotted vector specifies the desired pole location $e^{2\pi d/L}$ (FFT length *L*) from the given delay *d*.

Figure 7 denotes the estimated $\tilde{h}\left[n\right]$ (normalized) with 20 dB SNR and 20 ensemble average length for various ToF distributions. The desired delays *d* in Equation (57) are scheduled from 50 to 200 with 10 sample intervals and illustrated with various colors in Figure 7. The individual peak in the figure $\tilde{h}\left[n\right]$ distribution represents the simulation with given delay *d*. The non-parametric HD shows the near zero values below the 40-sample delay and the maximum peak delivers the exactly expected delay position with prominence. The wideband noise is observed over the whole range of samples above 40 samples in low profile. Note that the minimum phase realization can only deliver the clean and sharp abruption around the preferred window position. See Appendix A for further information.

The parametric HDs provide the poles and zeros from the signal models given in Equations (8) and (10). The second column in Figure 7 presents the pole-zero plots to deliver the pole locations with x mark and zero locations with o mark. The big circle in the pole-zero plot indicates the unit circle and the green dotted vector specifies the desired pole location from the given delay *d*. Note that the pole closer to the unit circle represents the peaky response on the evaluation as shown in Equations (50), (52), and (54). Therefore, the pole toward the unit circle is desirable for corresponding delay *d* position as $e^{2\pi d/L}$ where *L* is the FFT length. The prominent location in the parametric HD response is consistent with the green dotted vector. The closeness of the pole to the unit circle determines the sharpness of the response.

The parametric HD with Yule-walker method demonstrates the peak value at the desired position and the low hill points around 400 samples. The pole-zero plot shows the pole locations only since the Yule-walker method is derived from AR model as Equation (8) which contains the denominator coefficients for poles. The zeros are fixed in plot origin. The caution should be exercised for parametric methods in this paper because of the coefficient number sets. As we can observe the suspiciousness in the pole-zero plot, the coefficients are complex numbers. The real number coefficients present the symmetric poles and zeros distribution. However, the symmetric pole-zero location produces the biased estimation for low sample delays due to the mutual correlations. The green lines in pole-zero plot indicate the desired time delay locations and one pole of the Yule-walker method follows the green line direction. The other pole is placed and clustered in the perpendicular angle to reduce the correlation.

Similar to the Yule-walker method, the parametric HD with Prony method provides the peak location pointing the desired delay position in Figure 7. The low hill also can be detected with less consistency in position. The Prony method is derived from the ARMA model which includes the numerator and denominator for zeros and poles, respectively. The zero positions in the Prony method are distributed widely around the plot origin; therefore, the zeros barely contribute the magnitude modifications. Also, one pole of the Prony method follows the green line direction. The other pole is placed and clustered in the perpendicular angle to reduce the correlation as well.

The parametric HD with Steiglitz-McBride method shows sharp peak for desired delay position. Not only prominent peak but also low base values depict the best estimation configuration in HD algorithms. The Steiglitz-McBride method is also derived from the ARMA model. One pole is closely approached toward the unit circle in the pole-zero plot to deliver the sharp peak. The location of green lines and poles are well coordinated to indicate the proper time delay. Since the one pole contributes to the magnitude control significantly, the other pole and zeros are positioned widely in scattered manner. Observe that the poles in the Steiglitz-McBride method do not cross the unit circle for a stable response.

**Figure 8:**
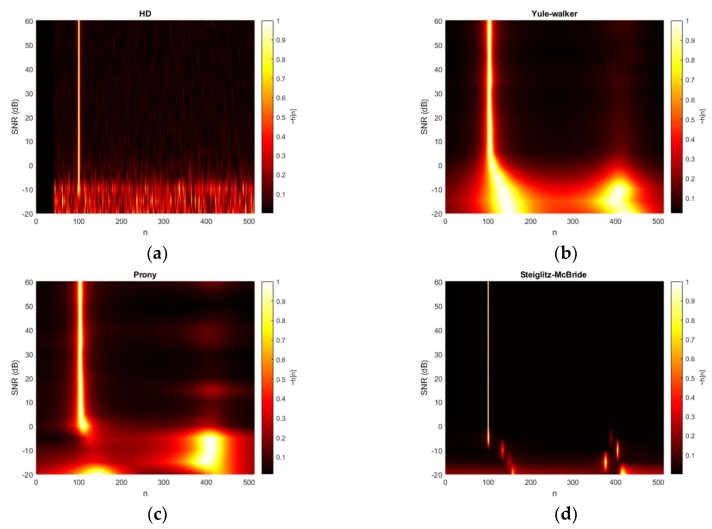
Estimated $\tilde{h}\left[n\right]$ distribution (normalized) with given SNR & 20 ensemble average from: (**a**) non-parametric HD top-view (interpolated); (**b**) Yule-walker HD top-view (interpolated); (**c**) Prony HD top-view (interpolated); (**d**) Steiglitz-McBride HD top-view (interpolated).

Figure 8 demonstrates the HD algorithm distributions for various SNR situations. The second microphone is positioned at 100-sample delay and the ensemble average length is arranged at 20 frames. The 2D plot in top viewpoint is generated by interpolation based on the simulated data for smooth texture. The non-parametric HD represents the peaky response below the 0 dB and the consistent response beyond the 0 dB. The single line in the 2D plot indicates the time delay in samples. The Yule-Walker and Prony algorithms also illustrate the weak performance below 0 dB condition with second strong peak around 400 samples. The consistent outputs are similarly examined beyond the 0 dB; however, the thickness of the pinnacle is wider than the non-parametric HD outcome. Therefore, certain bias and variance are expected in statistical performance for Yule-Walker and Prony method. The Steiglitz-McBride provides the thin line with low base values in Figure 8. The performance below 0 dB SNR is degraded as pointing to the improper locations.

**Figure 9:**
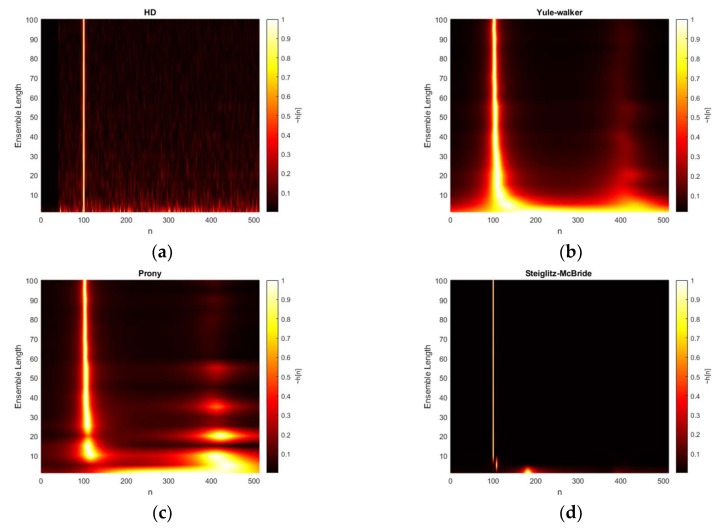
Estimated $\tilde{h}\left[n\right]$ distribution (normalized) with given ensemble average length & 0 dB SNR from: (**a**) non-parametric HD top-view (interpolated); (**b**) Yule-walker HD top-view (interpolated); (**c**) Prony HD top-view (interpolated); (**d**) Steiglitz-McBride HD top-view (interpolated).

Figure 9 presents the HD algorithm distributions for various ensemble average length conditions. The second microphone is located at 100-sample delay and the SNR is prescribed at 0 dB. The non-parametric HD represents the peaky response for short ensemble length and the consistent response for overall situations. Note that the steep cliff below 40-sample delay is caused by the FILF window. The Yule-walker and Prony also demonstrate the weak performance below 20 frames in ensemble length with second strong peak around 400-sample delay. The Yule-Walker algorithm delivers the coherent performance improvement in terms of mainlobe and sidelobe profile for increased ensemble length. However, the Prony algorithm provides the intermittent performance fluctuation up to the 40 ensemble length. The thickness of the dominant peak in Yule-walker and Prony method indicates the narrower outline for the increased ensemble length; therefore, the statistical performance by bias and variance are expected to be enhanced gradually. The Steiglitz-McBride denotes the very fine line with low base values in Figure 9. The performance degradation is only observed for one and five frame lengths for ensemble average.

**Figure 10:**
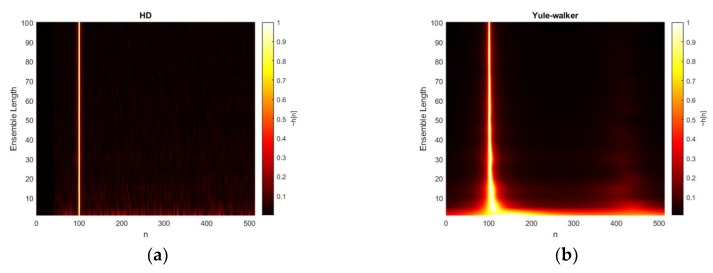

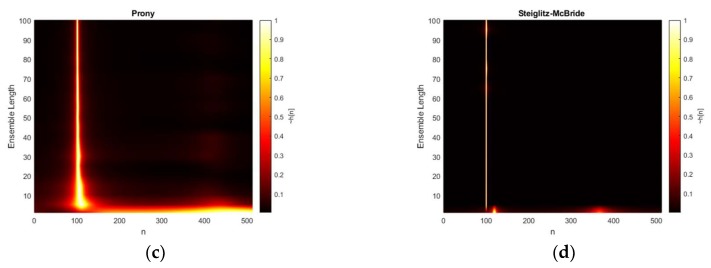
Estimated $\tilde{h}\left[n\right]$ distribution (normalized) with given ensemble average length & 20 dB SNR from: (**a**) non-parametric HD top-view (interpolated); (**b**) Yule-walker HD top-view (interpolated); (**c**) Prony HD top-view (interpolated); (**d**) Steiglitz-McBride HD top-view (interpolated).

Figure 10 shows the extension of previous ensemble length simulation for high SNR scenario. The all conditions are identical except the 20 dB SNR. The non-parametric HD depicts the consistent response for overall situations including the non-average situation. The Yule-Walker and Prony present the narrow mainlobe without significant degradation except single-frame processing situation. The mainlobe thickness in Yule-Walker and Prony method produces the narrower profile for the increased ensemble length as well. No intermittent performance fluctuation is observed in the Prony method except non-averaging processing. The Steiglitz-McBride method represents the laser line with low base values in Figure 10. The performance degradation is only observed for one frame length for ensemble average.

**Figure 11:**
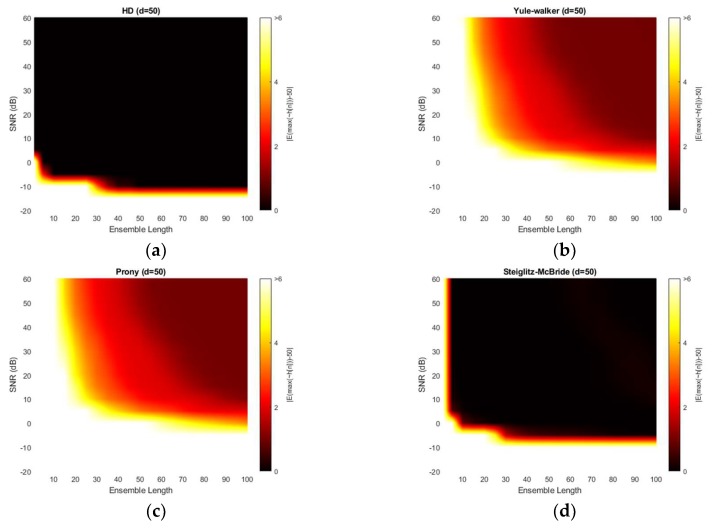
Average estimation error with given ensemble average length & SNR for 50-sample delay: (**a**) non-parametric HD; (**b**) Yule-walker HD; (**c**) Prony HD; (**d**) Steiglitz-McBride HD.

Figure 11 illustrates the absolute estimation error for 50-sample delay with various SNR and ensemble length. The non-parametric and Steiglitz-McBride method show the widespread dark black area which indicates the near zero bias. For near microphone placement (*d* = 50), the non-parametric and Steiglitz-McBride method provide the reduced biased estimation above the 0 dB SNR and 10 frame ensemble average. However, the Yule-Walker and Prony method represent the relatively high estimation error even for the high SNR and long average length. The observed least bias is around 0.5 sample in absolute value.

**Figure 12:**
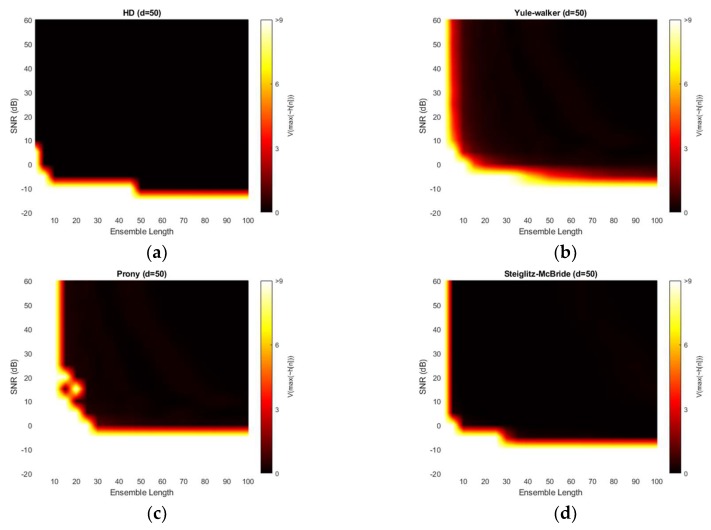
Variance of estimation with given ensemble average length & SNR for 50-sample delay: (**a**) non-parametric HD; (**b**) Yule-walker HD; (**c**) Prony HD; (**d**) Steiglitz-McBride HD.

Figure 12 demonstrates the variance for 50-sample delay with various SNR and ensemble length. Note that the variance above the 9 is normalized for investigating the small-scale distribution. All four methods provide the near zero variance for high SNR and long average length. This observation is consistent with the previous simulation outcomes which correspond to the mainlobe thickness. From a variance perspective, the non-parametric and Steiglitz-McBride method present a similarly reliable performance. The Prony method describes the least performance due to the inconsistent performance.

**Figure 13:**
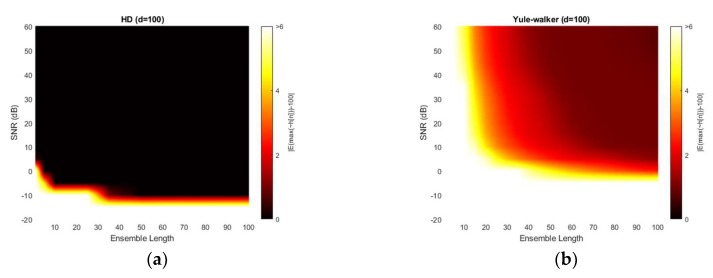

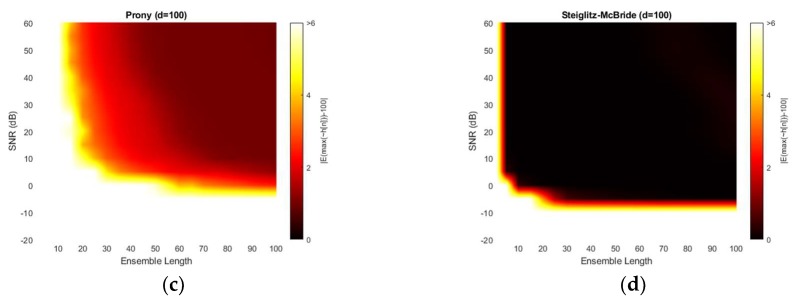
Average estimation error with given ensemble average length & SNR for 100-sample delay: (**a**) non-parametric HD; (**b**) Yule-walker HD; (**c**) Prony HD; (**d**) Steiglitz-McBride HD.

Figure 13 illustrates the absolute estimation error for 100-sample delay with various SNR and ensemble length. No significant differences are observed in the estimation bias from 50-sample delay performance. The non-parametric and Steiglitz-McBride method present the widespread near zero bias. The Yule-Walker and Prony method denote the relatively high estimation error for overall situations. The observed least bias is still around 0.5 sample in absolute value for middle distance microphone location (*d* = 100).

**Figure 14:**
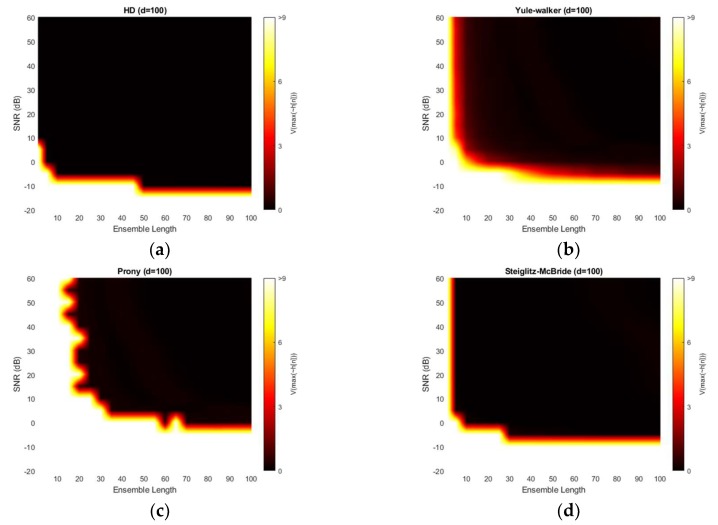
Variance of estimation with given ensemble average length & SNR for 100-sample delay: (**a**) non-parametric HD; (**b**) Yule-walker HD; (**c**) Prony HD; (**d**) Steiglitz-McBride HD.

Figure 14 depicts the variance for 100-sample delay with various SNR and ensemble length. Similar to the 50-sample delay simulation, all four methods provide the near zero variance for high SNR and long average length. Except the Prony method, the variance distribution is almost identical to the near microphone simulation (*d* = 50) counterpart. The Prony method demonstrates the wider white region and further uneven edges for high variance. The Prony method delivers the worse performance in middle distance microphone location (*d* = 100) for variance perspective.

**Figure 15:**
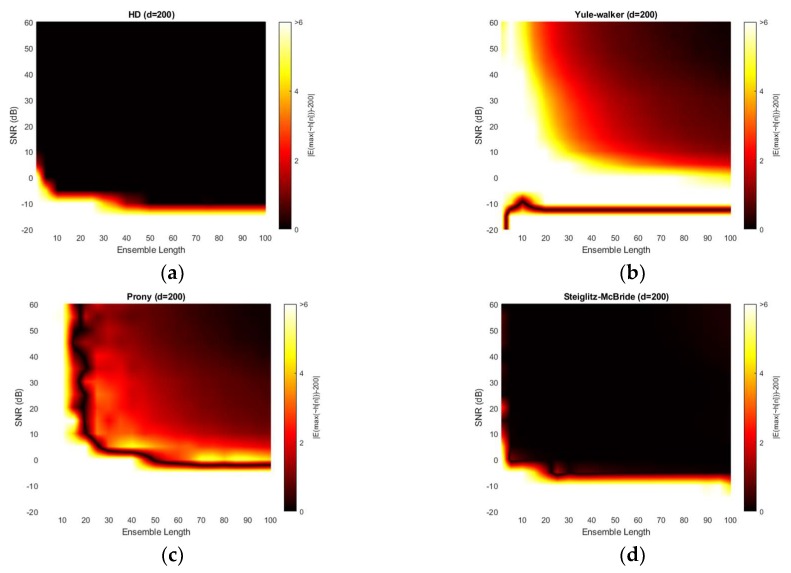
Average estimation error with given ensemble average length & SNR for 200-sample delay: (**a**) non-parametric HD; (**b**) Yule-walker HD; (**c**) Prony HD; (**d**) Steiglitz-McBride HD.

**Figure 16:**
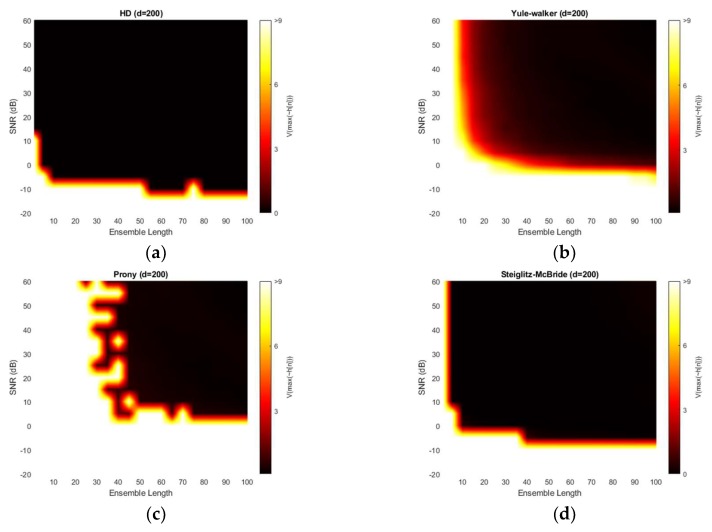
Variance of estimation with given ensemble average length & SNR for 200-sample delay: (**a**) non-parametric HD; (**b**) Yule-walker HD; (**c**) Prony HD; (**d**) Steiglitz-McBride HD.

Figure 15 presents the absolute estimation error for 200-sample delay with various SNR and ensemble length. The non-parametric and Steiglitz-McBride method illustrate the consistent performance for far microphone location (*d* = 200) as well. No considerable differences are visible from previous simulation outcomes. However, the Yule-Walker and Prony methods provide lower bias for the high SNR and long average length compare to the pervious simulations. Also, the black line is perceived in Yule-Walker and Prony methods. The local low bias does not signify good estimation performance because of the high variance in the given area as shown in Figure 16.

Figure 16 shows the variance for 200-sample delay with various SNR and ensemble length. Similar to the previous simulation outcomes, all four methods provide the near zero variance for high SNR and long average length. Except the Yule-Walker and Prony method, the variance distribution is almost identical to the near and middle microphone simulation counterpart. The Yule-walker method produces the slightly increased white area. The Prony method illustrates the wider and uneven area for high variance. The bias distribution from Figure 15 exhibits the decreased bias river in the low SNR and/or short average length area which corresponds to the high variance region. The reliable estimator requires the low bias as well as low variance. 

## 4. Results

**Figure 17:**
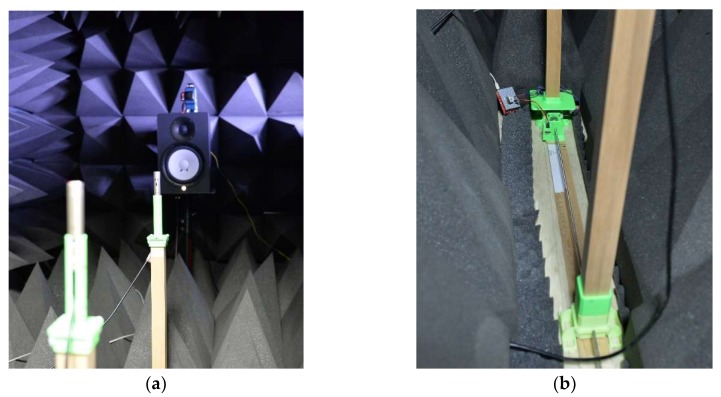
Acoustic experiment in the anechoic chamber: (**a**) two microphones & speaker configuration with laser guidance; (**b**) automatic relocation system to control microphone distance.

The acoustic experiments are performed and evaluate in an anechoic chamber that has been validated to demonstrate partial conformance with ISO 3745 [37] for the 250 Hz–16 kHz one-third octave band in a free-field chamber and for the 1–16 kHz one-third octave band in a hemi-free-field chamber [30]. The non-parametric and parametric HD algorithms are analyzed with the free-field chamber mode, which contains fully covered surfaces for all directions with acoustic wedges. Observe that the experiment configuration indicates the two microphones and one source with proper distance, and the HD algorithms denote the forward and inverse real cepstrum algorithm with or without parametric estimations. As shown in Figure 17, the directional alignment for microphones and transmitter is guided by the line laser (GLL 3-80 P, Bosch, Gerlingen, Germany) located above the sound source speaker. The first microphone is located at the direct-front direction 1.00 m away from the speaker. The distance between the microphones is controlled by the automatic relocation system, shown in Figure 17, based on the ball screw and stepping motor. The linear motion from the ball screw repositions the second microphone via employing the microprocessor (MSP430F5529LP, Texas Instruments, Dallas, TX, USA) based open loop control. The plastic parts are realized by the 3D printer (Replicator 2, MakerBot, Brooklyn, NY, USA) from polylactic acid (PLA) filament as illustrated in Figure 17. 

The MATLAB programming operates the microphones (C-2, Behringer, Tortola, British Virgin Islands), computer-connected audio device (Quad-Capture, Roland, Hamamatsu, Japan), analog mixer (MX-1020, MPA Tech, Seoul, Republic of Korea), speaker (HS80M, Yamaha, Hamamatsu, Japan) and automatic relocation system simultaneously. The MATLAB system object with the audio stream input/output (ASIO) driver processes the real-time audio in terms of generation, reception, and execution. In the designated distance, the audio is recorded for 20 s with 48 kHz sampling rate. The first and last one second data is discarded to reduce the interruption by computational and environmental conditions. Therefore, the overall 18 s of recorded data is utilized for individual distance experiments. The individual data frame is organized by 1024 samples and the new ensemble average process is initiated after the 10 frames later. The rest of the frames are overlapped for data consistency.

In order to coordinate the experiments and simulations, the physical distance is converted for the discrete samples to measure the ToF. The resolution (samples/centimeter) Δ can be calculated from the sampling frequency and sound speed (at 25 °C) as shown below.

(61) $$\Delta =\frac{f_{s}}{c}=\frac{48000}{34613}=1.3868\;\left(\frac{\mathrm{samples}}{\mathrm{centimeter}}\right)@\;25℃$$

The recorded distances are arranged from 20 to 80 in 10 cm intervals as shown in Table 1. The corresponding distance in samples are computed by multiplying the resolution with the physical distances. Note that the calculated values are rounded to the nearest integer value. If there is one sample error in ToF estimation, 0.7211 cm (1/Δ)  distance is away from the true position. The measurement accuracy is subject to the environment parameters such as temperature and sound speed.

**Figure 18:**
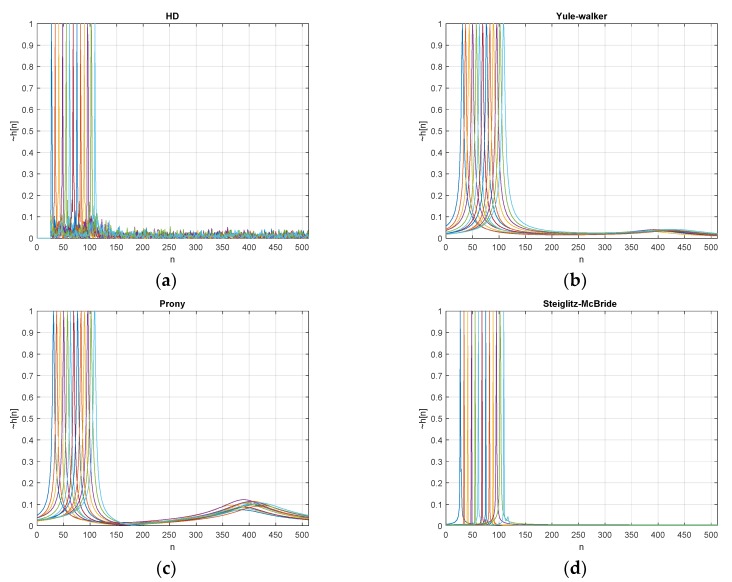
Estimated $\tilde{h}\left[n\right]$ distribution (normalized) with 100 ensemble average for ToF (20~80 cm in 10 cm interval) from: (**a**) non-parametric HD; (**b**) Yule-walker HD; (**c**) Prony HD; (**d**) Steiglitz-McBride HD.

In the experiments, the FILF window *w*[*n*] is decided to eliminate the 25 sample below and maximum phase realization. Observe that the computed non-parametric and parametric HD output $\tilde{h}\left[n\right]$ is normalized. Figure 18 denotes the estimated $\tilde{h}\left[n\right]$ with 100 ensemble average length for given ToF situations. The non-parametric HD demonstrates the near zero values below the 25-sample delay and the maximum peak represents the expected delay position with distinction. The Yule-Walker method presents the peak at the desired position and the minor hill around 400 samples. The Prony method provides the peak location pointing to the desired delay. The slightly increased hill can be identified with a certain inconsistency in position. The Steiglitz-McBride method illustrates sharp peak for desired delay position with low base values. 

Table 2 provides the statistical performance for short ensemble average length as 20 frames. The non-parametric and Steiglitz-McBride method shows the low bias and variance in overall. The consistent discrepancy between the measurements and estimations are detected at 55, 97 and 111 sample distance in target. We assume that the estimated values are correct in the situations because of the susceptible measurement conditions. The Yule-Walker and Prony methods represent the high bias with variance. The majority situation shows the consistent high estimation error due to the low variance except the Prony at 111 sample distance. A single iteration of the 83 Prony results generates the significant second mainlobe for estimation around 400 sample distance which contributes to the high variance. 

Table 3 demonstrates the statistical performance for middle ensemble average length as 50 frames. The non-parametric HD delivers the zero bias and zero variance based on the assumption in previous paragraph. The Yule-Walker and Prony methods produce the substantially reduced bias and variance than the short ensemble length counterparts. For both methods, the estimation biases exhibit high values in low time delay conditions and the variances depict the coherent values for entire range. The variance abruption in Prony method is removed and stabilized in 50 ensemble length. The Steiglitz-McBride method establishes the similar performance as the short ensemble length with minor degradation at 28- and 97-sample delay cases. 

Table 4 delivers the statistical performance for long ensemble average length as 100 frames. The non-parametric and Steiglitz-McBride illustrate the zero bias and zero variance. The Yule-Walker and Prony method generate the improved bias and stabilized variance. The mean value of both methods further approaches the measured number than the previous experiments. Therefore, long ensemble average length provides the better statistical performance for non-parametric and parametric HD algorithms. Observe that the 100-frame ensemble length is equivalent to the 2.1333 s data.

The introduced non-parametric and parametric HD algorithms well derive the ToF information in most of situations. Note that the output of non-parametric HD in this experiment is represented by 1024 samples. However, the output of Prony HD and Steiglitz-McBride HD is denoted by 5 coefficients as *a*_1,_ *a*_2_, *b*_0_, *b*_1_, and *b*_2_ according to the ARMA model shown in Equation (10). Also, the output of Yule-Walker HD is indicated by two coefficients as *a*_1_ and *a*_2_ along with the AR model demonstrated in Equation (8). The given length of output will be served as the featured information for complete SSL system based on the machine learning (or deep learning) in future works. 

## 5. Conclusions

This paper presents a novel time of flight estimation method based on the non-parametric and parametric homomorphic deconvolutions. The non-parametric homomorphic deconvolution is realized by the forward and inverse real cepstrum with frequency-invariant linear filtering. The proper window configuration removes the low time delay components and maximum phase realization. The numerical distribution of non-parametric homomorphic deconvolution produces the likelihood of time of flight information between the two microphones. Therefore, the time location corresponding to the maximum value indicates the estimation. The parametric methods replace the last inverse Fourier transform of non-parametric homomorphic deconvolution by parametric estimation algorithms as Yule-walker, Prony, and Steiglitz-McBride. The derived complex number coefficients for the parametric method properly specify the time of flight position by maximum value. Observe that the parametric methods require the evaluation process for numerical distribution. The simulations for various signal-to-noise ratio and ensemble average length present non-statistical and statistical performance outcomes. The non-parametric homomorphic deconvolution and Steiglitz-McBride methods illustrate the consistent distribution with low bias and variance overall. The Yule-Walker and Prony methods are vulnerable to the simulation conditions. The high signal-to-noise ratio and long ensemble length generally denote the better statistical performance in both methods. The experiments in anechoic chamber also delivers the comparable results as the simulation. The increased ensemble length shows the zero bias and variance for non-parametric homomorphic deconvolution and Steiglitz-McBride method. The Yule-Walker and Prony methods display the performance improvement in terms of bias and variance for longer length conditions. 

Sound localization for the transport is a challenging topic because of the complexity induced by the structure and environment. This paper builds upon the fundamentals for sound localization by time of flight estimation based on non-parametric and parametric homomorphic deconvolution. Future works will offer optimal microphone array configuration with homomorphic deconvolution algorithm to maximize the localization performance by using the machine learning and deep learning approaches. Non-parametric homomorphic deconvolution is appropriate for the applying the deep learning method. The parametric methods are designed for the machine learning algorithms because the features are extracted by signal modelling and corresponding coefficients. This paper verifies the algorithm functionality by statistical simulations and experiments. However, the algorithm performance could be appended by the different perspective from machine/deep learning algorithms. In other words, the least performance method in this paper could be proved for improved realization by chance. The extensive simulation and experiments are required to obtain the receiver structure, homomorphic deconvolution, estimation order, learning method, and etc. Note that the problem size by receiver number is not correlated to the localization system complexity at all. Future papers will discuss the feasible localization system in detail based on this article outcome.

## Figures and Tables

**Table 1:** Conversion table for target distance in samples at 25 °C.

**Target Distance** **(cm)**	20	30	40	50	60	70	80
**Target Distance (Samples)**	28	42	55	69	83	97	111

**Table 2:** Experiment results as mean (upper row) and variance (lower row) for 20 ensemble length.

**Target**	28	42	55	69	83	97	111
**HD**	28	42	56	69	83	96.01	110
	0	0	0	0	0	0.0100	0
**Yule-walker**	34.96	47.57	61.28	73.34	86.52	99.18	111.57
	0.4499	0.5413	0.4467	0.2751	0.4478	0.4182	0.7852
**Prony**	34.98	47.57	61.27	73.25	86.45	98.99	115.01
	0.4628	0.5413	0.4655	0.2889	0.3476	0.4023	1164.0364
**Steiglitz-McBride**	28.94	42	56	69	83	96.67	110
	0.0573	0	0	0	0	0.2220	0

**Table 3:** Experiment results as mean (upper row) and variance (lower row) for 50 ensemble length.

**Target**	28	42	55	69	83	97	111
**HD**	28	42	56	69	83	96	110
	0	0	0	0	0	0	0
**Yule-walker**	32.59	45.25	59.01	71.05	84.36	97.22	109.96
	0.2454	0.1899	0.1138	0.0481	0.2593	0.2019	0.2391
**Prony**	32.63	45.26	59	71.03	84.38	97.13	109.91
	0.2373	0.1960	0.1013	0.0247	0.2373	0.1867	0.2581
**Steiglitz-McBride**	29.39	42	56	69	83	96.42	110
	1.3543	0	0	0	0	0.7285	0

**Table 4:** Experiment results as mean (upper row) and variance (lower row) for 100 ensemble length.

**Target**	28	42	55	69	83	97	111
**HD**	28	42	56	69	83	96	110
	0	0	0	0	0	0	0
**Yule-walker**	31.84	44.49	58.04	70.08	83.69	96.55	109.33
	0.1362	0.2533	0.0389	0.0746	0.2155	0.2512	0.2252
**Prony**	31.85	44.53	58.04	70.07	83.75	96.36	109.25
	0.1268	0.2523	0.0389	0.0631	0.1917	0.2335	0.1917
**Steiglitz-McBride**	28	42	56	69	83	96	110
	0	0	0	0	0	0	0

**Figure A1:**
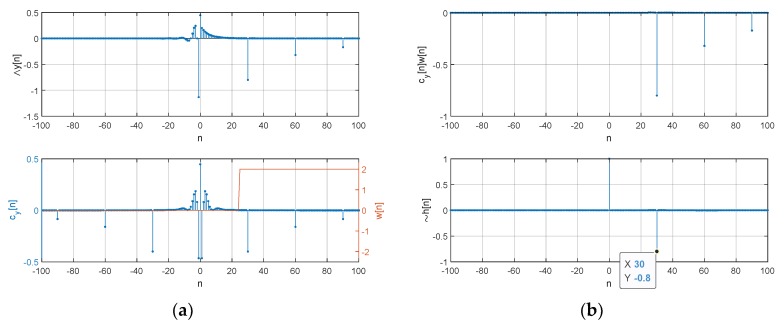
HD computational procedures for numerical example: (**a**) Complex cepstrum (above) and real cepstrum (below); (**b**) FILF output (above) and estimated propagation function (below).

## Appendix A

The equations below present the non-parametric HD based on the real cepstrum in *z* domain. The received signal *Y*(*z*) is arrived at two receivers with time delay *d* samples as shown in *H*(*z*). The sound source *X*(*z*) is described by poles $\alpha_{k}$ and zeros $\beta_{k}$ inside the unit circle as well as the zeros $\gamma_{k}$ outside the unit circle.

(A1) $$\begin{array}{c} Y\left(z\right)=X\left(z\right)H\left(z\right)=\frac{A\prod_{k=1}^{M_{i}}\left(1-\beta_{k}z^{-1}\right)\prod_{k=1}^{M_{o}}\left(1-\gamma_{k}^{-1}z^{-1}\right)}{\prod_{k=1}^{N}\left(1-\alpha_{k}z^{-1}\right)}\left(1-\mu z^{-d}\right)\\ \left|\alpha_{k}\right|<1,\;\left|\beta_{k}\right|<1,\;\left|\gamma_{k}\right|<1,\;\left|\mu \right|<1\;\; \end{array}$$

Organize the equation into that below to represent the sequences composed of a stable sum exponential.

(A2) $$\begin{array}{c} Y\left(z\right)=\frac{A\prod_{k=1}^{M_{i}}\left(1-\beta_{k}z^{-1}\right)\prod_{k=1}^{M_{o}}\gamma_{k}^{-1}z^{-M_{o}}\left(\gamma_{k}z-1\right)}{\prod_{k=1}^{N}\left(1-\alpha_{k}z^{-1}\right)}\left(1-\mu z^{-d}\right)\\ =\frac{Az^{-M_{o}}\prod_{k=1}^{M_{o}}\left(-\gamma_{k}^{-1}\right)\prod_{k=1}^{M_{i}}\left(1-\beta_{k}z^{-1}\right)\prod_{k=1}^{M_{o}}\left(1-\gamma_{k}z\right)}{\prod_{k=1}^{N}\left(1-\alpha_{k}z^{-1}\right)}\left(1-\mu z^{-d}\right) \end{array}$$

Factorize into a product of minimum phase and maximum phase signals as below.

(A3) $$Y\left(z\right)=z^{-M_{o}}X_{min}\left(z\right)X_{max}\left(z\right)H\left(z\right)$$

(A4) $$X_{min}\left(z\right)=\frac{A\prod_{k=1}^{M_{i}}\left(1-\beta_{k}z^{-1}\right)}{\prod_{k=1}^{N}\left(1-\alpha_{k}z^{-1}\right)}$$

(A5) $$X_{max}\left(z\right)=\prod_{k=1}^{M_{o}}\left(-\gamma_{k}^{-1}\right)\prod_{k=1}^{M_{o}}\left(1-\gamma_{k}z\right)$$

Apply logarithm of *Y*(*z*) is below. The product of terms is transformed to the sum of logarithmic terms.

(A6) $$\begin{array}{c} \log\left(Y\left(z\right)\right)=\hat{Y}\left(z\right)=\log\left|A\right|+\log\left(z^{-M_{o}}\right)+\sum_{k=1}^{M_{o}}\log\left|\gamma_{k}^{-1}\right|+\sum_{k=1}^{M_{i}}\log\left(1-\beta_{k}z^{-1}\right)\\ +\overset{M_{o}}{\underset{k=1}{\sum }}\log\left(1-\gamma_{k}z\right)+\log\left(1-\mu z^{-d}\right)-\overset{N}{\underset{k=1}{\sum }}\log\left(1-\alpha_{k}z^{-1}\right) \end{array}$$

The Taylor series for the natural logarithm is given below known as Mercator series [38].

(A7) $$\log\left(1-z\right)=-\overset{\infty }{\underset{n=1}{\sum }}\frac{z^{n}}{n}\;\;\;\mathrm{for}\;\left|z\right|<1$$

Employ the Mercator series for each logarithmic term as below.

(A8) $$-\overset{\infty }{\underset{m=1}{\sum }}\frac{\beta_{k}^{m}}{m}\delta \left[n-m\right]\overset{z}{↔}\log\left(1-\beta_{k}z^{-1}\right)=-\overset{\infty }{\underset{m=1}{\sum }}\frac{\beta_{k}^{m}z^{-m}}{m}\;\mathrm{for}\;\left|z\right|>\left|\beta_{k}\right|$$

(A9) $$-\overset{\infty }{\underset{m=1}{\sum }}\frac{\gamma_{k}^{m}}{m}\delta \left[n+m\right]\overset{z}{↔}\log\left(1-\gamma_{k}z\right)=-\overset{\infty }{\underset{m=1}{\sum }}\frac{\gamma_{k}^{m}z^{m}}{m}\;\mathrm{for}\;\left|z\right|<\left|\gamma_{k}^{-1}\right|$$

The inverse *Z*-transform from the Mercator series provides the complex cepstrum $\hat{y}\left[n\right]$ as below. It is important to note that the magnitude of each term is decreased by power of poles/zeros at least as fast as 1/|n|. However, the term induced by time delay *d* is decaying at the rate of 1/|n| in every *d* sample stride. Therefore, the $\hat{h}\left[n\right]$ represents significantly slow decline rate.

(A10) $$\begin{array}{c} \hat{y}\left[n\right]=\left\{\log\left|A\right|+\overset{M_{o}}{\underset{k=1}{\sum }}\log\left|\gamma_{k}^{-1}\right|\right\}\delta \left[n\right]+\overset{\infty }{\underset{m=1}{\sum }}\left(-\overset{M_{i}}{\underset{k=1}{\sum }}\frac{\beta_{k}^{m}}{m}+\overset{N}{\underset{k=1}{\sum }}\frac{\alpha_{k}^{m}}{m}\right)\delta \left[n-m\right]\;\\ -\overset{\infty }{\underset{m=1}{\sum }}\frac{\mu^{m}}{m}\delta \left[n-dm\right]-\overset{\infty }{\underset{m=1}{\sum }}\left(\overset{M_{o}}{\underset{k=1}{\sum }}\frac{\gamma_{k}^{m}}{m}\right)\delta \left[n+m\right] \end{array}$$

The relation between the real cepstrum $c_{y}\left[n\right]$ and complex cepstrum $\hat{y}\left[n\right]$ is below.

(A11) $$c_{y}\left[n\right]=\frac{\hat{y}\left[n\right]+\hat{y}\left[-n\right]}{2}$$

The derived real cepstrum is below.

(A12) $$\begin{array}{c} c_{y}\left[n\right]=\left\{\log\left|A\right|+\overset{M_{o}}{\underset{k=1}{\sum }}\log\left|\gamma_{k}^{-1}\right|\right\}\delta \left[n\right]+\frac{1}{2}\overset{\infty }{\underset{m=1}{\sum }}\left(-\overset{M_{i}}{\underset{k=1}{\sum }}\frac{\beta_{k}^{m}}{m}+\overset{N}{\underset{k=1}{\sum }}\frac{\alpha_{k}^{m}}{m}\right)\delta \left[n\mp m\right]\;\\ -\frac{1}{2}\overset{\infty }{\underset{m=1}{\sum }}\frac{\mu^{m}}{m}\delta \left[n\mp dm\right]-\frac{1}{2}\overset{\infty }{\underset{m=1}{\sum }}\left(\overset{M_{o}}{\underset{k=1}{\sum }}\frac{\gamma_{k}^{m}}{m}\right)\delta \left[n\pm m\right] \end{array}$$

The FILF is defined as below. The window function *w*[*n*] simply removes the undesired low sample delays (below *l* in this example) as well as maximum phase realization which is represented by the anti-causal components. Note that the HD based on the DFT denotes the high half of the sequence as the anti-causal components due to the circular property of the DFT.

(A13) $$\hat{h}\left[n\right]=c_{y}\left[n\right]w\left[n\right]$$

(A14) w[n]=2u[n−l]  where l≤d

After the FILF, the slow decaying exponential sequence is survived from the filtering. The anti-causal components are eliminated by the window as well.

(A15) $$\hat{h}\left[n\right]\approx -\overset{\infty }{\underset{m=1}{\sum }}\frac{\mu^{m}}{m}\delta \left[n-dm\right]$$

Observe as below that the other sequences are decreasing the magnitude at the rate of 1/|n| for rapid converging.

(A16) $$\frac{1}{2}\overset{\infty }{\underset{m=1}{\sum }}\left(-\overset{M_{i}}{\underset{k=1}{\sum }}\frac{\beta_{k}^{m}}{m}+\overset{N}{\underset{k=1}{\sum }}\frac{\alpha_{k}^{m}}{m}\right)\delta \left[n-m\right]\approx 0\;\;for\;m\geq l$$

(A17) $$-\frac{1}{2}\overset{\infty }{\underset{m=1}{\sum }}\left(\overset{M_{o}}{\underset{k=1}{\sum }}\frac{\gamma_{k}^{m}}{m}\right)\delta \left[n-m\right]\approx 0\;\;for\;m\geq l$$

Apply the *Z*-transform and use the Mercator series to combine into the logarithmic term as below.

(A18) $$\hat{H}\left(z\right)\approx -\overset{\infty }{\underset{m=1}{\sum }}\frac{\mu^{m}z^{-dm}}{m}=-\overset{\infty }{\underset{m=1}{\sum }}\frac{{\left(\mu z^{-d}\right)}^{m}}{m}=\log\left(1-\mu z^{-d}\right)$$

Employ the exponential function over the given $\hat{H}\left(z\right)$ to obtain the simple polynomial.

(A19) $$e^{\hat{H}\left(z\right)}\approx e^{\log\left(1-\mu z^{-d}\right)}=\left(1-\mu z^{-d}\right)$$

The inverse *Z*-transform provides the estimated propagation function $\tilde{h}\left[n\right]$ as below.

(A20) $$\tilde{h}\left[n\right]\approx \delta \left[n\right]-\mu \delta \left[n-d\right]$$

In this paper, the last computational stage of HD algorithm is realized by forward transformation instead of inverse transformation as shown below. Therefore, the final stage of HD can be selected from FFT, Yule-walker, Prony, and Steiglitz-McBride algorithms for non-parametric and parametric methods.

(A21) $$\tilde{h}\left[n\right]\overset{\mathrm{FFT}}{↔}e^{\hat{H}\left[k\right]}\;\mathrm{for}\;N=2^{k}\;\;\;k\in \mathbb{N}$$

The implementation of inverse transformation based on the forward can be derived as below. The conjugated input to the forward transformation generates the conjugated inverse transformation with scale.

(A22) $$\tilde{h}\left[n\right]=\frac{1}{N}\overset{N-1}{\underset{k=0}{\sum }}e^{\hat{H}\left[k\right]}e^{j\frac{2\pi }{N}kn}=\frac{1}{N}{\left\{\overset{N-1}{\underset{k=0}{\sum }}{\left(e^{\hat{H}\left[k\right]}\right)}^{*}e^{-j\frac{2\pi }{N}kn}\right\}}^{*}=\frac{1}{N}{\left\{{\mathrm{FFT}}_{N}{\left(e^{\hat{H}\left[k\right]}\right)}^{*}\right\}}^{*}$$

The magnitude of the HD algorithm is required to determine the ToF. Therefore, the absolute value of the output is given as below.

(A23) $$\left|\tilde{h}\left[n\right]\right|=\frac{1}{N}\left|{\mathrm{FFT}}_{N}{\left(e^{\hat{H}\left[k\right]}\right)}^{*}\right|$$

Below is the example by numbers. The sound source *X*(*z*) consists of poles and zeros at 0.9, 0.7, $1.25e^{-j\frac{\pi }{4}}$, $1.25e^{j\frac{\pi }{4}}$. The propagation function *H*(*z*) shows the time delay at 30 samples with 0.8 magnitude decreasing.

$$Y\left(z\right)=X\left(z\right)H\left(z\right)=\frac{\left(1-0.7z^{-1}\right)\left(1-1.25e^{-j\frac{\pi }{4}}z^{-1}\right)\left(1-1.25e^{j\frac{\pi }{4}}z^{-1}\right)}{\left(1-0.9z^{-1}\right)}\left(1-0.8z^{-30}\right)$$

Organize the equation to represent the sequences composed of a stable sum exponential.

$$Y\left(z\right)=\frac{\left(1-0.7z^{-1}\right)1.25^{2}z^{-2}\left(1-0.8e^{j\frac{\pi }{4}}z\right)\left(1-0.8e^{-j\frac{\pi }{4}}z\right)}{\left(1-0.9z^{-1}\right)}\left(1-0.8z^{-30}\right)$$

The corresponding real cepstrum is below. Note that the maximum phase realization is represented by anti-causal components.

$$\begin{array}{c} \hat{y}\left[n\right]=2\log\left(1.25\right)\delta \left[n\right]+\overset{\infty }{\underset{m=1}{\sum }}\overset{\infty }{\underset{m=1}{\sum }}\left(-\frac{0.7^{m}}{m}+\frac{0.9^{m}}{m}\right)\delta \left[n-m\right]\\ -\overset{\infty }{\underset{m=1}{\sum }}\frac{0.8^{m}}{m}\delta \left[n-30m\right]-\overset{\infty }{\underset{m=1}{\sum }}\left(\frac{0.8^{m}e^{j\frac{\pi }{4}m}}{m}+\frac{0.8^{m}e^{-j\frac{\pi }{4}m}}{m}\right)\delta \left[n+m\right] \end{array}$$

The real cepstrum is below based on the above equation.

$$\begin{array}{c} c_{y}\left[n\right]=2\log\left(1.25\right)\delta \left[n\right]+\frac{1}{2}\overset{\infty }{\underset{m=1}{\sum }}\left(-\frac{0.7^{m}}{m}+\frac{0.9^{m}}{m}\right)\delta \left[n\mp m\right]\\ -\frac{1}{2}\overset{\infty }{\underset{m=1}{\sum }}\frac{0.8^{m}}{m}\delta \left[n\mp 30m\right]-\overset{\infty }{\underset{m=1}{\sum }}\left(\frac{0.8^{m}}{m}\cos\left(\frac{\pi }{4}m\right)\right)\delta \left[n\pm m\right] \end{array}$$

Apply the FILF as below.

$$\hat{h}\left[n\right]=c_{y}\left[n\right]w\left[n\right]$$

The window function *w*[*n*] is designed to remove the time delay below 25 samples which maintains the given propagation function *h*[*n*].

w[n]=2u[n−l]  where l≤d, l=25, d=30

Employ the FFT for inverse cepstrum as below.

$$\hat{H}\left[k\right]={\mathrm{FFT}}_{512}\left(-\frac{1}{2}\overset{3}{\underset{m=1}{\sum }}\frac{0.8^{m}}{m}\delta \left[n-30m\right]\right)$$

Use the exponential function and FFT to obtain the final HD output.

$$\tilde{h}\left[n\right]=\frac{1}{N}{\left\{{\mathrm{FFT}}_{512}{\left(e^{\hat{H}\left[k\right]}\right)}^{*}\right\}}^{*}$$

Figure A1 demonstrates the computational procedures for given numerical example above. The complex cepstrum illustrates the causal component from minimum phase realization and anti-causal component from maximum phase realization. The real cepstrum combines the complex cepstrum with time reflection for even symmetric distribution. Also, the window function *w*[*n*] and FILF output are also depicted to remove the sound source *x*[*n*]. The HD output $\tilde{h}\left[n\right]$ estimates the time delay by investigating the distribution.
